# Taxonomic revision of the genus *Xenopholis* Peters, 1869 (Serpentes: Dipsadidae): Integrating morphology with ecological niche

**DOI:** 10.1371/journal.pone.0243210

**Published:** 2020-12-11

**Authors:** Daniel Faustino Gomes, Josué Azevedo, Roberta Murta-Fonseca, Søren Faurby, Alexandre Antonelli, Paulo Passos

**Affiliations:** 1 Departamento de Vertebrados, Museu Nacional, Universidade Federal do Rio de Janeiro, Rio de Janeiro, Brasil; 2 Department of Biological and Environmental Sciences, University of Gothenburg, Gothenburg, Sweden; 3 Gothenburg Global Biodiversity Centre, University of Gothenburg, Gothenburg, Sweden; 4 Coordenação de Biodiversidade, Programa de Coleções Científicas Biológicas, Instituto Nacional de Pesquisas da Amazônia, Amazonas, Brazil; 5 Laboratório de Zoologia, Campus do Pantanal, Universidade Federal de Mato Grosso do Sul, Bairro Universitário, Corumbá, Mato Grosso do Sul, Brasil; 6 Royal Botanic Gardens, Kew, Surrey, United Kingdom; 7 Department of Plant Sciences, University of Oxford, Oxford, United Kingdom; Laboratoire de Biologie du Développement de Villefranche-sur-Mer, FRANCE

## Abstract

A reliable identification and delimitation of species is an essential pre-requisite for many fields of science and conservation. The Neotropical herpetofauna is the world’s most diverse, including many taxa of uncertain or debated taxonomy. Here we tackle one such species complex, by evaluating the taxonomic status of species currently allocated in the snake genus *Xenopholis* (*X*. *scalaris*, *X*. *undulatus*, and *X*. *werdingorum*). We base our conclusions on concordance between quantitative (meristic and morphometric) and qualitative (color pattern, hemipenes and skull features) analyses of morphological characters, in combination with ecological niche modeling. We recognize all three taxa as valid species and improve their respective diagnosis, including new data on color in life, pholidosis, bony morphology, and male genitalia. We find low overlap among the niches of each species, corroborating the independent source of phenotypic evidence. Even though all three species occur in the leaf litter of distinct forested habitats, *Xenopholis undulatus* is found in the elevated areas of the Brazilian Shield (Caatinga, Cerrado and Chaco), whereas *X*. *scalaris* occurs in the Amazon and Atlantic rainforests, and *X*. *werdingorum* in the Chiquitanos forest and Pantanal wetlands. We discuss the disjunct distribution between Amazonian and Atlantic Forest snake species in the light of available natural history and ecological aspects. This study shows the advantages of combining multiple data sources for reliable identification and circumscription of ecologically similar species.

## Introduction

The dipsadid snake genus *Xenopholis* Peters 1869 constitutes a monophyletic group that comprises three species: *Xenopholis scalaris* (Wucherer, 1862), *X*. *undulatus* (Jensen, 1900) and *X*. *werdingorum* Jansen, Álvarez and Köhler, 2009 [1, 2]. It includes small to moderate-sized snakes (300–450 mm), with cryptozoic lifestyle (i.e., underneath soil surface and high humidity habitats) [3–6], feeding primarily on anurans [4, 7–9], but occasionally also lizards [6]. Members of the genus are widely distributed in the cis-Andean Neotropics: *Xenopholis scalaris* ranges from the cis-Andean portion of South America along the ombrophilous forests of Colombia, Guianas, Ecuador, Peru, Bolivia and Brazil, with disjunct populations along the Atlantic Forest [4, 7–16]; *Xenopholis undulatus* is distributed in Paraguay and also in open areas of the Brazilian Shield, from Maranhão south to Paraná [13]; and *Xenopholis werdingorum* occurs at the Chiquitanos forests of Bolivia and within the Pantanal wetlands [5, 13, 17, 18]. Due to their small body sizes and secretive lifestyle, species of this genus are rarely found [3, 7], resulting in poor representation in herpetological collections and scarce literature regarding their biology and morphological variation.

Here we evaluate the taxonomic status of species currently allocated in the genus *Xenopholis* (*X*. *scalaris*, *X*. *undulatus*, and *X*. *werdingorum*) on the basis of concordance between quantitative (meristic and morphometric) and qualitative (hemipenial and skull features) analyses of morphological characters, in combination with niche overlap analyses. From the resulting refined locality records, we produced environmental niche modeling for gaining insights on the determinants of species distributions in this genus under current and past environmental conditions.

## Taxonomic résumé

Wucherer [10] described *Elapomorphus scalaris* based on two specimens from Canavieiras (15°39’S 38°57’W; 5m above sea level, hereafter asl) and Mata de São João (12°32’S 38°18’W; 31m asl), state of Bahia, Brazil. Peters [19] erected the genus *Xenopholis* in order to accommodate *Elapomorphus scalaris*. Boulenger [20] synonymized *Xenopholis braconnieri* Peters [19] and *Gerrhosteus prosopis* Cope [21] with *Xenopholis scalaris* based on a specimen from “brasilien” (= Brazil) and two specimens from Nauta in the Peruvian Amazonia, respectively. Werner [22] named *Sympeltophis ungalioides* based on an individual from central Brazil. Peters and Orejas-Miranda [23] placed this last taxon in the synonymy of *Xenopholis scalaris*. Jensen [24] described *Oxyrhopus undulatus* based on a specimen from Lagoa Santa (19°38'S 43°53'W; 835m asl), state of Minas Gerais, Brazil. Schenkel [25] named *Paroxyrhopus reticulatus* based on a specimen from “Bemalcue” (= Bernal-Cué, 25°15'S 57°17’W; 218m asl), Paraguay. Werner [26] described *Oxyrhopus latifrontalis* based on a specimen collected in the east of Minas Gerais, Brazil. Amaral [27] described *Paroxyrhopus atropurpureus* based on an individual collected at Nova Baden (19°58’S 44°6’W; 848m asl), state of Minas Gerais, Brazil. Amaral [28] placed *O*. *latifrontalis* and *P*. *atropurpureus* in the synonymy of *P*. *undulatus*. Peters and Orejas-Miranda [23] recognized *P*. *undulatus* and *P*. *reticulatus* as valid species. Hoge and Federsoni [29] proposed the synonymy of the latter two names and transferred *Oxyrhopus undulatus* to the genus *Xenopholis* due to unique vertebral morphology with neural spines expanded, forming rugose shields. Jansen [5] described *Xenopholis werdingorum* based on three specimens from Santa Cruz de la Sierra, department of Santa Cruz, Bolivia. The author diagnosed *X*. *werdingorum* from *X*. *undulatus* mainly based on differences in dorsal coloration.

## Material and methods

### Material and techniques for phenotypic characters

We examined 349 *Xenopholis* specimens including: 261 *Xenopholis scalaris*, 76 *X*. *undulatus*, and 12 *X*. *werdingorum* housed at 20 herpetological collections. The institutional abbreviations are as detailed in Sabaj [30].

The terminology used for the cephalic scales follows Jansen, Álvarez and Köhler [5], while the counting of ventral and subcaudal scales are based on Dowling [31]. We measured most variables with an analogical caliper DIGIMESS^®^ to the nearest 0.01 mm, except for snout-vent and tail lengths, which were taken with a millimetric ruler to the nearest 1.0 mm. We examined maxillary teeth in situ under stereoscope through a narrow lateromedial incision between the supralabials and the maxillary arch. After removing tissues covering the maxillary bone, we counted the teeth and the empty sockets of specimens preserved in alcohol. We determined the sex of specimens by checking for the presence of the hemipenes through a ventral incision at the base of the ventral surface of the tail. We defined mature specimens through inspection of convoluted deferent ducts in males, and the occurrence of vitellogenic follicles (at least 5 mm in length) [32], eggs or pleated glandular uterus in females [33]. We prepared hemipenes according to the method for the preparation of preserved hemipenes modified from Pesantes [34], by replacing KOH for distilled water [35]. Prior to the inflation with petroleum jelly, the organs remained 15 minutes in alcohol solution of Alizarin red to stain the ornamented calcareous structures, according to an adaptation from the original procedure used by Uzzell [36] and modified by Harvey and Embert [37]. Terminology for hemipenial descriptions follows Dowling and Savage [38] and Zaher [39]. We examined osteological features through μCT Scan high-resolution images (*Xenopholis scalaris*, MNRJ 17070 and UMMZ 245078; *Xenopholis undulatus* UMMZ 108820; *Xenopholis werdingorum*, UFMT-R 12051) and dried skulls (*Xenopholis undulatu*s, MNRJ 18728). We scanned specimens with a high energy μCT Scan Skyscan 1176/Bruker system at COPPE, Instituto Alberto Cruz Coimbra de Pós-Graduação e Pesquisa de Engenharia, Universidade Federal do Rio de Janeiro, Rio de Janeiro, Brazil. We reconstructed the images using the FDK algorithm [40] with the software InstaRecon version 1.3.9.2, and analyzed the results with the software CTVox version 2.7.0. We accessed μCT from UMMZ specimens through MorphoSource Project <morphosource.org> (*X*. *scalaris* M20632-39127; *X*. *undulatus* M42465-76525). We prepared dried skulls following the techniques modified from Hangay and Dingley [41]. We followed Cundall and Irish [42] for osteological terminology.

### Geographical data

Coordinates of localities were acquired by consulting the original data available in museum catalogs, digital databases, or geographical gazetteers (e.g., IBGE, 2011). We refined, whenever possible, the origin of records obtained from the literature or in museum databases without specific field coordinates using Google Earth Pro 7.1.2 (Google, 2005). We include literature data only when the information was sufficient to ensure the unequivocal identification of the species.

### Species concept

In this study, we followed the unified species concept from de Queiroz [43, 44]. We considered the presence of one or more exclusive apparently fixed diagnostic characters (either morphological or ecological), which distinguishes a given taxon from the others as a species delimitation criterion. Nonetheless, as the sample sizes assessed here were in some cases too small for statistical tests of qualitative characters [45], we looked for concordance between discrete and continuous characters, as well as corroboration from environmental niche modeling. The correspondence between these kinds of data might represent independent evidence for species delimitation. However, in the cases of discrete characters, we explicitly searched for congruence with additional lines of evidence to increase the confidence for diagnosing [46, 47].

### Operational analytical units

We divided the available sample into four groups based on the current taxonomy and considering the disjunct pattern of distribution of *Xenopholis scalaris*. The operational analytical categories are: Group 1 = *Xenopholis scalaris* from the Atlantic Forest; Group 2 = *X*. *scalaris* from the Amazon Basin; Group 3 = *X*. *undulatus*, and Group 4 = *X*. *werdingorum*. For strictly exploratory analytical purposes, we further divided the populations of *Xenopholis scalaris* to investigate if there is any additional level of phenotypic differentiation. We considered Atlantic Forest populations as a single group due to the relatively small sample available and divided the Amazonian samples in two operation units: a Guiana Shield group, with locations north of the Amazon River and west of the Negro River; and south of the Amazon River within the Amazon drainage basin; see Henderson [48] for a similar analytical approach.

### Quantitative analyses

To reduce the ontogenetic bias in the morphometric analyses, we selected only adult (supposedly mature specimens; see above) specimens to compose the dataset. As we did not find information about sexual maturity for the genus in the literature, we performed a small incision above the cloaca of the specimens to delimit the smallest mature specimen (details above).

We evaluated the assumptions of univariate normality and homoscedasticity with Kolmogorov-Smirnov and Levene tests, respectively [49]. In cases where the distributions of the characters violated such assumptions, we performed non-parametric tests or excluded such variables from the analyses [49]. We performed analyses of univariate (ANOVA) and multivariate (MANOVA) variance in order to test for the presence or absence of sexual dimorphism within each group [50]. We found evidence of sexual dimorphism in some groups; therefore, we performed parametric tests separately for males and females. We also performed discriminant function analyses (DFA) with 95% confidence from an exploratory perspective to evaluate the quantitative discrimination between currently recognized species. Specimens and variables with missing data above 30% were discarded from the statistical analyses [46]. The remaining missing data were substituted by the ingroup mean for each variable with the function "replace missing data" [51]. All the statistical tests were performed in Statistica 5.1 [51].

### Qualitative analyses

We selected the following variables for the population frequency analyses: (1) dorsal color with spots forming discontinuous lateral bands along the body (e.g., *X*. *scalaris*), (2) irregular spots forming winding vertebral stripe (e.g., *X*. *undulatus*), (3) absence of spots with a nearly uniform dorsal coloration (e.g., *X*. *werdingorum*); (4) presence of well-defined or inconspicuous vertebral line (e.g., *X*. *scalaris*).

Hemipenes were analyzed as follows: hemipenial body regarding the general shape of capitulum (capitulum shorter than the hemipenial body vs. capitulum approximately of similar length than the hemipenial body); sulcus spermaticus bifurcation (outside capitulum vs. within the capitulum); level of lateral expansion of the sulcus spermaticus (nearly centrolineal vs. centripetal); capitulum ornamentation (spinulate vs. papillate calyces); arrangement (serial [linearly or transversally] vs. irregular) of hooked spines on the sulcate, lateral and asulcate sides of hemipenial body; and ornamentation of medium-distal portion of hemipenial body on the asulcate side (nude vs. ornamented with papillae or spines).

### Niche modeling and niche overlap

We generated model predictions for *Xenopholis scalaris* and *X*. *undulatus* with ensemble forecasting modeling [52]. Ensemble modeling integrates properties of algorithms of different complexities, generally yielding higher prediction accuracy [53]. We obtained the final ensemble models for each species by applying an AUC-weighting (Area Under the Curve) to the results of 15 different modeling algorithms available in the *sdm* R-package (S1 Table—[54]). The performance of the models were assessed using 5-fold cross-validation (10 replications), totaling 150 models per species. The predictive performance of the final ensembled models was measured using True Skill Statistics—TSS [55]. Due to the small number of known localities for *X*. *werdingorum* (N = 8), we used an ensembling of small models technique [56]. For this, models were produced with all possible combinations of only two environmental variables each time and then weighted by AUC. For the small models, we used only four different modeling algorithms (S1 Table), as modeling performance in this technique does not increase with the use of additional algorithms [56]. We recognize that the low number of presence records makes the model for *X*. *werdingorum* less reliable than for the others; therefore, we carefully interpret the results for this species.

To produce the models, we randomly generated 148 pseudo-absences, which is equivalent to the total number of presence records for all *Xenopholis* species at the chosen resolution (see below). *Xenopholis* species present low detectability levels, precluding the use of target-group bias corrections (e.g., selecting pseudo-absences only in well-sampled localities for all snakes species in South America, but in which *Xenopholis* species are absent). Therefore, we used the most recommendable approach in this case, and we randomly selected the pseudo-absences across the geographical extent of the analyses (among cells where *Xenopholis* species are absent) [56, 57]. As the species of the genus *Xenopholis* are mostly found in forested habitats, we delimited the extent area for sampling the pseudo-absences and the respective values of environmental layers (see below) to the region to east of the Andean mountains corresponding to the maximum latitudinal range of tropical forests since the Last Maximum Glacial [58], which encompasses all known records of *Xenopholis*. This was only done to incorporate environmental characteristics of areas in which species of this genus would probably be able to disperse (e.g., no clear single geographical barriers such as the Andean mountains).

We obtained ten temperature and nine precipitation bioclimatic layers from the CHELSA project [59]. The scarcity of climatic stations in the Neotropical region is known to decrease modeling performance [60]. However, CHELSA variables are estimated from both climatic stations and from models of atmospheric circulation, which improves climate predictions for isolated areas. We also downloaded soil variables from soilgrids.org [61], including the percentage of clay and sand—median values for the first 15 cm of the soil surface—and elevation data from GMTED 2010 [62]. We aggregated by median and projected all layers to an equal-area Berhmann projection with a resolution equivalent to 0.2 x 0.2° at the 30th degree of latitude [63]. This resolution is adequate for modelling the distribution of species at a semi-continental scale and for smoothing the effects of potential georeferencing errors, especially derived from old museum specimens (e.g., georeferenced at municipality scales).

As very little is known about specific habitat requirements in *Xenopholis*, to select the most relevant environmental variables for each species, we run a first round of models using the Random Forests algorithm in the *sdm* R-package [54, 64]. We ran models with all possible combinations of three variables each time (N = 1.540), calculating the most important variables using an AUC-based permutation—median values per variable [65]. From this result, we chose the first six more important variables with no multicollinearity problems (Variance Inflation Factor < 10) [66], except for elevation. Elevation is an indirect predictor of species niche, and therefore, high response values for this variable may indicate that additional environmental determinants are missing for the targeted species [67]. Due to the small number of records, for *X*. *werdingorum*, we performed the Random Forests variable selection by modeling all combinations with two variables each time. Finally, we also projected the distribution of the species to the Last Glacial Maximum (Community Climate System Model 4, LGM– 22,000 years ago).

We tested the degree of niche overlap in the environmental space for each pair of species using the Schoener’s D metric [68], which goes from zero (no overlap) to one (total overlap). For this, we used the PCA-env approach to produce a reduced two-dimensional linear representation of all 22 variables described above [69]. To test the significance of the overlap, we used two different randomizations. In the first one, occurrence records of each pair of species are shuffled 100 times (niche equivalence). In the second, the whole density of occurrence records of one of the species pairs (calculated as part of the PCA-env approach) is randomly reallocated within the available climatic space 1,000 times. The significance is accessed by comparing the observed D metric with the distribution of the mentioned randomizations. All niche overlap tests were performed in R using the scripts provided by Broennimann [69]. We additionally tested niche overlap for the disjunct records of *X*. *scalaris* in Amazonia and the Atlantic Forest, to verify whether these populations are isolated not only in the geographical but also in the environmental space.

## Results

### Quantitative analyses

The analysis of the gonad maturation indicated the SVL of 207 mm for the smaller adult male in *Xenopholis*. Therefore, we considered specimens (males and females) ≥ 207 mm as adults for all subsequent statistical approaches. We found sexual dimorphism for *Xenopholis scalaris* and *Xenopholis undulatus* in the number of ventral scales, with females presenting higher values (F_220,2_ = 18.4; p <0.001); in the number of subcaudal scales, with males presenting higher values (F_220,2_ = 95.95, p <0.001); and in the number of preocular scales, with males presenting greater number (F_220,2_ = 4.50, p = 0.03). All other meristic and morphometric variables did not exhibit apparent secondary dimorphism. Due to the low sample size for *Xenopholis werdingorum* (N < 30), we performed a Mann-Whitney test that indicated dimorphism for the tail length in this species (U = 15; p < 0.02; N = 12).

**Fig 1:**
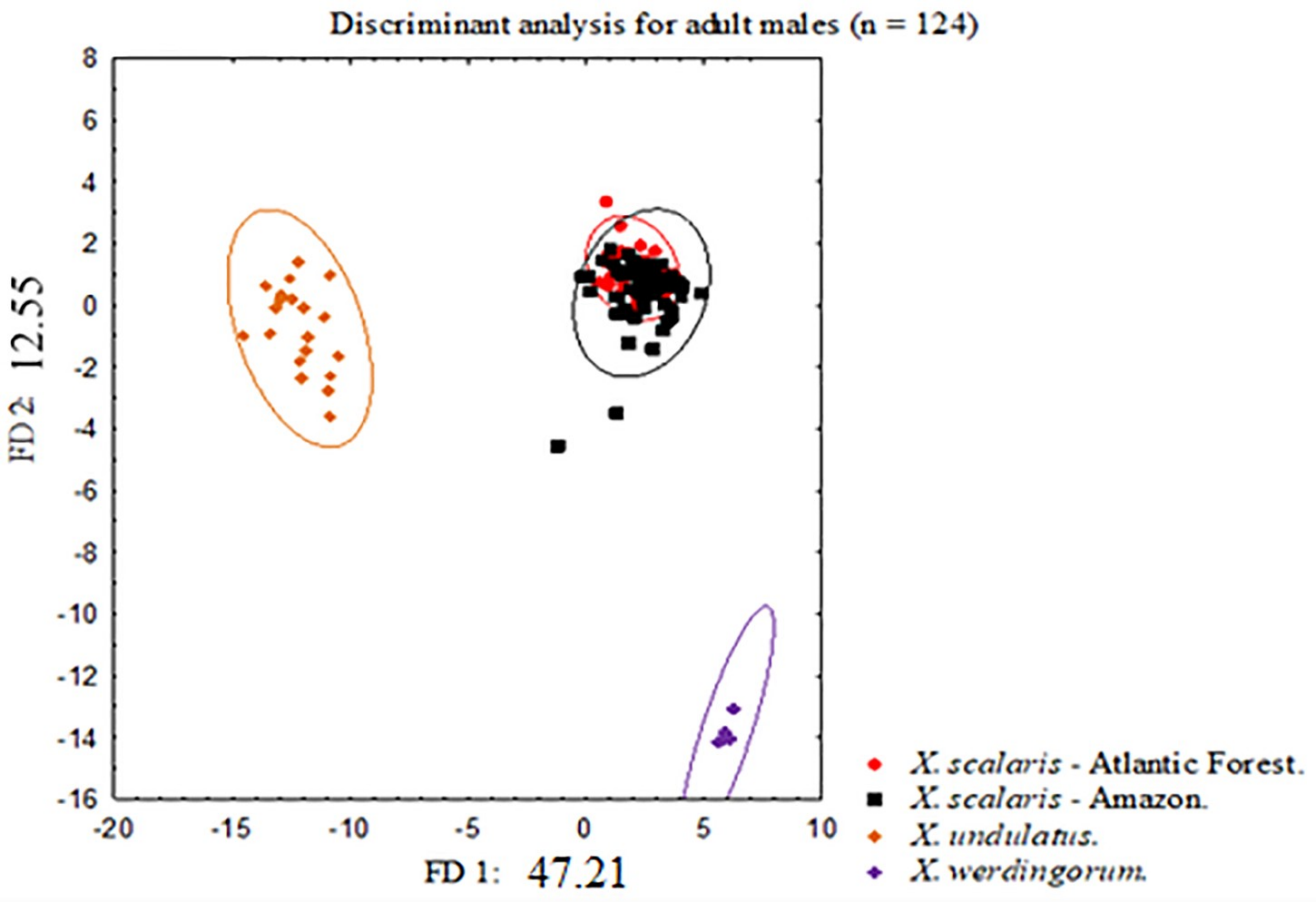
Bivariate plot derived from first two axes from scores of linear discriminant analyses performed for adult males (N = 124) of *Xenopholis scalaris*—Atlantic forest; *Xenopholis scalaris*—Amazon; *Xenopholis undulatus*, and *Xenopholis werdingorum*.

**Fig 2:**
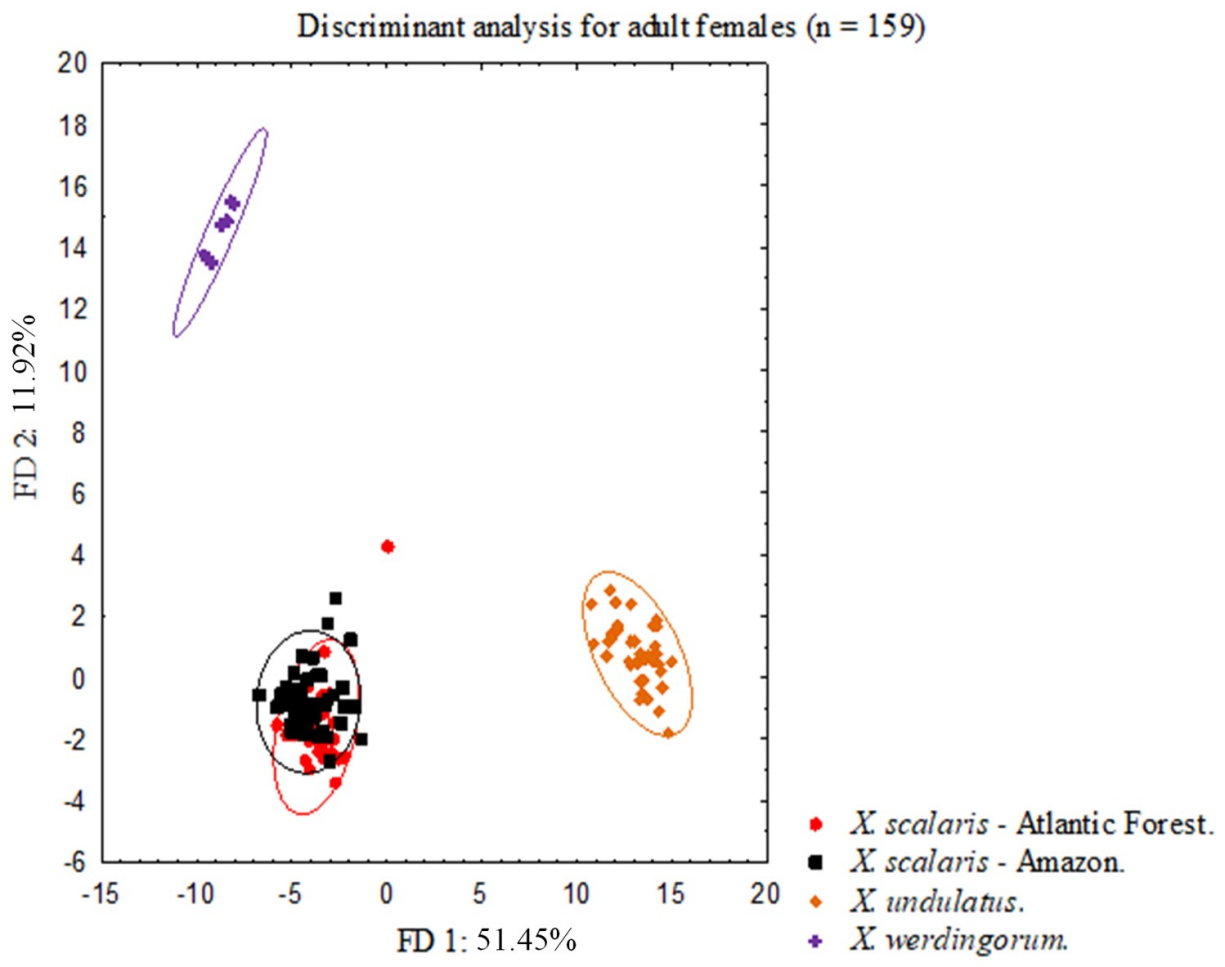
Bivariate plot derived from first two axes from scores of linear discriminant analyses performed for adult females (N = 159) of *Xenopholis scalaris*—Atlantic forest; *Xenopholis scalaris*—Amazon; *Xenopholis undulatus* and *Xenopholis werdingorum*.

The discriminant analyses performed for males (17 variables, N = 124) and females (17 variables, N = 159) showed that the disjunct set of populations of *Xenopholis scalaris* from Amazonia and Atlantic Forest entirely overlap in the 95% confidence intervals. In contrast, *X*. *undulatus* and *X*. *werdingorum* were completely discriminated between themselves and from the populations of *X*. *scalaris* (Figs 1 and 2). In DFA for males, the first DF was responsible for 47.21% of discrimination considering the predefined groups, while the second DF was responsible for 12.55% (Fig 1). In DFA for females, the first DF was responsible for 51.45% discrimination and the second for 11.92% (Fig 2).

**Fig 3:**
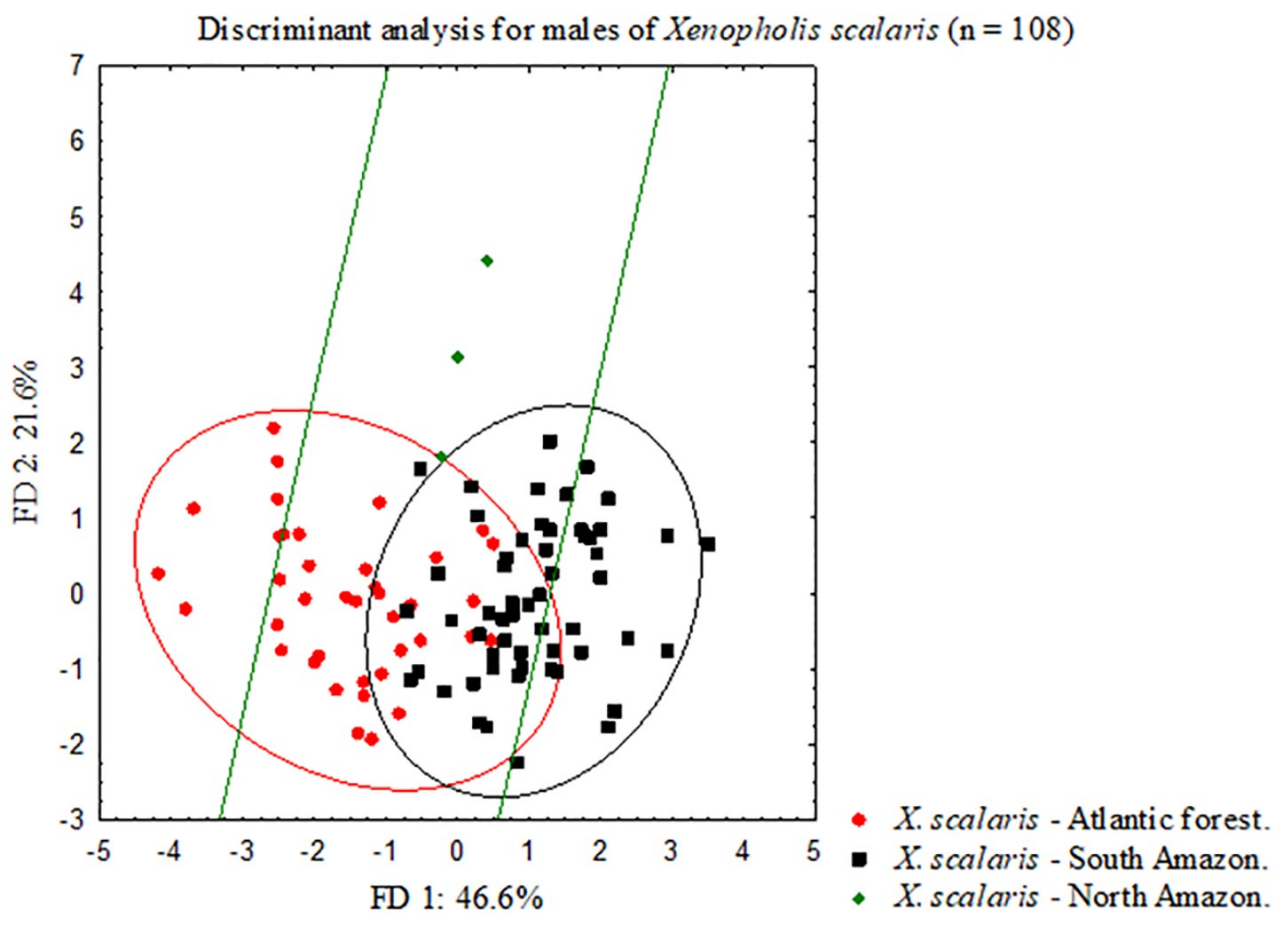
Bivariate plot derived from first two axes from scores of linear discriminant analyses performed for adult males (N = 108) from subpopulations of *Xenopholis scalaris*—Atlantic forest, North Amazon and South Amazon.

**Fig 4:**
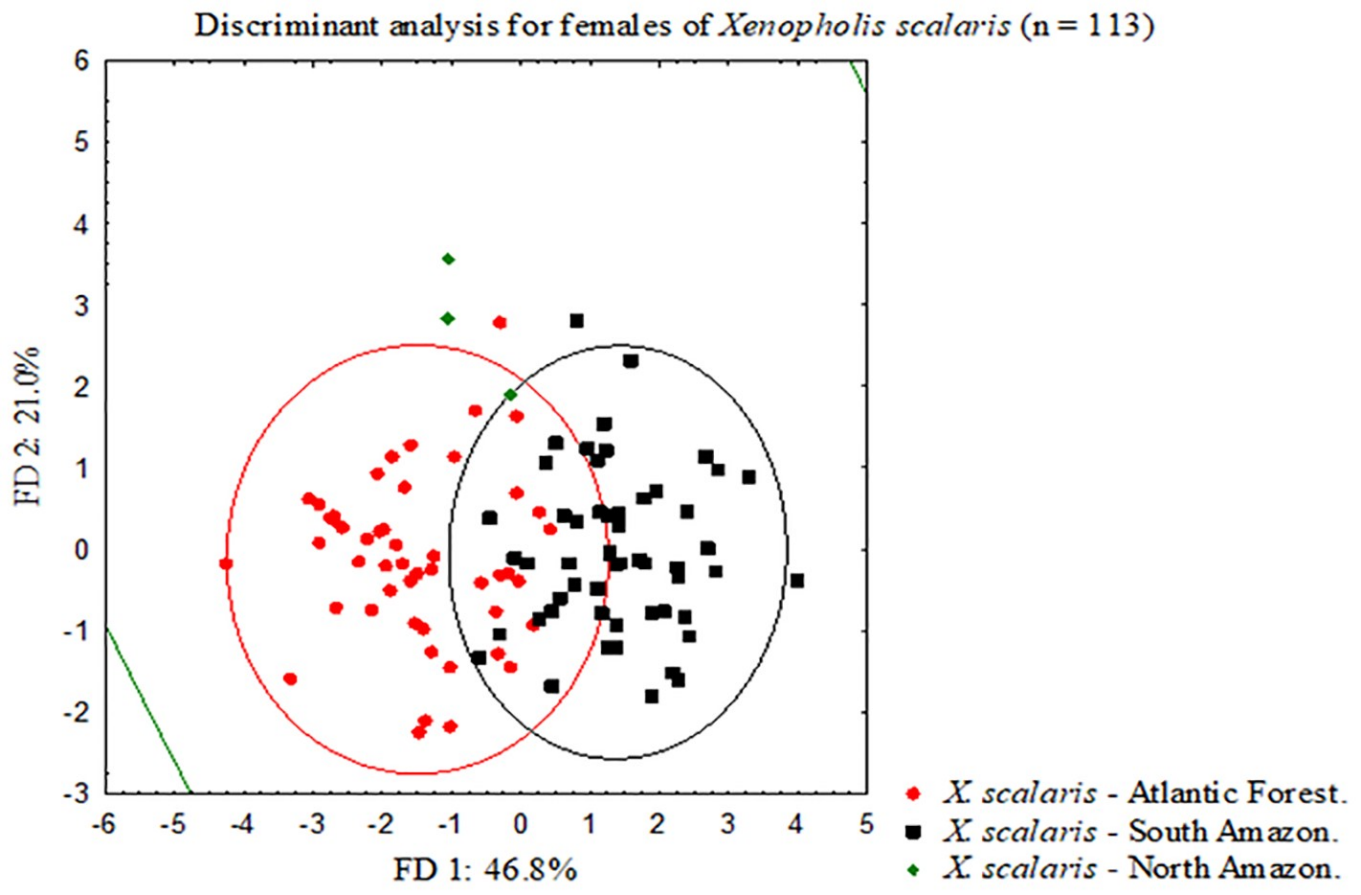
Bivariate plot derived from first two axes from scores of linear discriminant analyses performed for adult females (N = 113) from subpopulations of *Xenopholis scalaris*—Atlantic forest, North Amazon and South Amazon.

The additional discriminant analysis aiming to verify the segregation of Amazonian populations of *Xenopholis scalaris* resulted in a high degree of overlap considering the 95% confidence ellipses for each group in both analysis (males and females) (Figs 3 and 4). In synthesis, the Atlantic Forest and Amazonian populations of *Xenopholis scalaris* are not distinguishable from each other, as is the case between the north and south groups of Amazonian specimens (considering the Amazon River as a putative barrier for north/south dispersion). In contrast, *X*. *scalaris* as a single evolutionary unit is distinguished from *X*. *undulatus* and *X*. *werdingorum*, differing from them by the number of dorsal spots, ventral and subcaudal scales, number of anterior dorsal scales rows (from cervical region to midbody), and number of prefrontals (Table 1).

**Table 1:** Analysis of 95% confidence intervals: Group 1 (*Xenopholis scalaris*—Atlantic forest); Group 2 (*Xenopholis scalaris*—AM, South of the Amazon river and West of Rio Negro); Group 3 (*Xenopholis undulatus*), Group 4 (*Xenopholis werdingorum*) and Group 5 (*Xenopholis scalaris*—AM, Northern Amazon river and East Rio Negro).

		Dorsal spots	Ventral (M)	Ventral (F)	Subcaudals (M)	Subcaudals (F)	Dorsal I	Dorsal II	Prefrontal
**Group 1**	Min	28.00	126	128	29	27	17	17	1
	Max	41.00	144	151	41	36	17	17	1
	Mean	34.19	132.98	135.37	34.98	31.65	17	17	1
	Sd	2.61	4.66	4.36	2.44	2.26			0
	-95%	33.69	132	134	34	31			1
	+95%	34.69	134	136	36	32			1
	N	106	44	62	44	62	106	106	105
**Group 2**	Min	24.00	127	130	28	29	17	17	1
	Max	40.00	169	175	45	42	17	17	1
	Mean	32.27	136.66	142.90	36.53	32.48	17	17	1
	Sd	2.97	6.55	7.65	4.26	2.58			0
	-95%	31.77	135	141	36	32			1
	+95%	32.76	138	145	38	33			1
	N	139	70	69	70	69	139	139	126
**Group 3**	Min	35.00	160	168	36	33	19	19	2
	Max	79.00	190	196	55	60	19	19	2
	Mean	69.32	178.86	181.02	44.59	41.47	19	19	2
	Sd	5.90	7.04	6.36	5.53	5.22			0
	-95%	67.93	176	179	42	40			2
	+95%	70.72	182	183	47	43			2
	N	71	22	49	22	49	71	71	70
**Group 4**	Min		181	180	46	38	19	19	2
	Max		195	196	54	48	19	19	2
	Mean		190	187.14	49.50	42.57	19	19	2
	Sd		6.63	5.90	3.42	3.64	0	0	0
	N		4	7	4	7	11	11	11
**Group 5**	Min	28	131	133	31	29	17	17	1
	Max	32	137	142	32	32	17	17	1
	Mean	29.83	135	137.33	31.67	30.33	17	17	1
	Sd	1.47	3.46	4.51	0.58	1.53			
	N	6	3	3	3	3	6	6	6

Abbreviations: F = female; M = male; max = maximum; min = minimum; N = sample number; sd = standard deviation; -95% = lower limit of the 95% confidence interval, + 95% upper limit of the 95% confidence interval.

### Qualitative analyses

#### Color pattern

We divided *Xenopholis scalaris* into Atlantic Forest (N = 109) and Amazonian (N = 152) sets of populations. All specimens examined had discontinuous bands on the lateral region of the body, with ventral and supralabial scales uniformly white. The vertebral line was well defined (Atlantic Forest 59.6%, Amazonia 56.6%) or barely distinct (Atlantic Forest 40.4%, Amazonia 43.4%); dorsal ground color light brown (Atlantic Forest 29.4%, Amazonia 27.6%), brown (Atlantic Forest 50.5%, Amazonia 46.7%) or dark brown (Atlantic Forest 21.1%, Amazonia 25.6%); dorsum of head is light brown (Atlantic Forest 29.4%, Amazonia 27.6%), brown (Atlantic Forest 49.5%, Amazonia 46.7%) or dark brown (Atlantic Forest 21.1%, Amazonia 25.6%). As there are no obvious single geographical barrier separating subpopulations of *Xenopholis undulatus* and *X*. *werdingorum*, and both species present relatively conspicuous coloration through its entire distribution, we did not perform additional population frequency analyses for these species.

#### Hemipenial morphology

**Fig 5:**
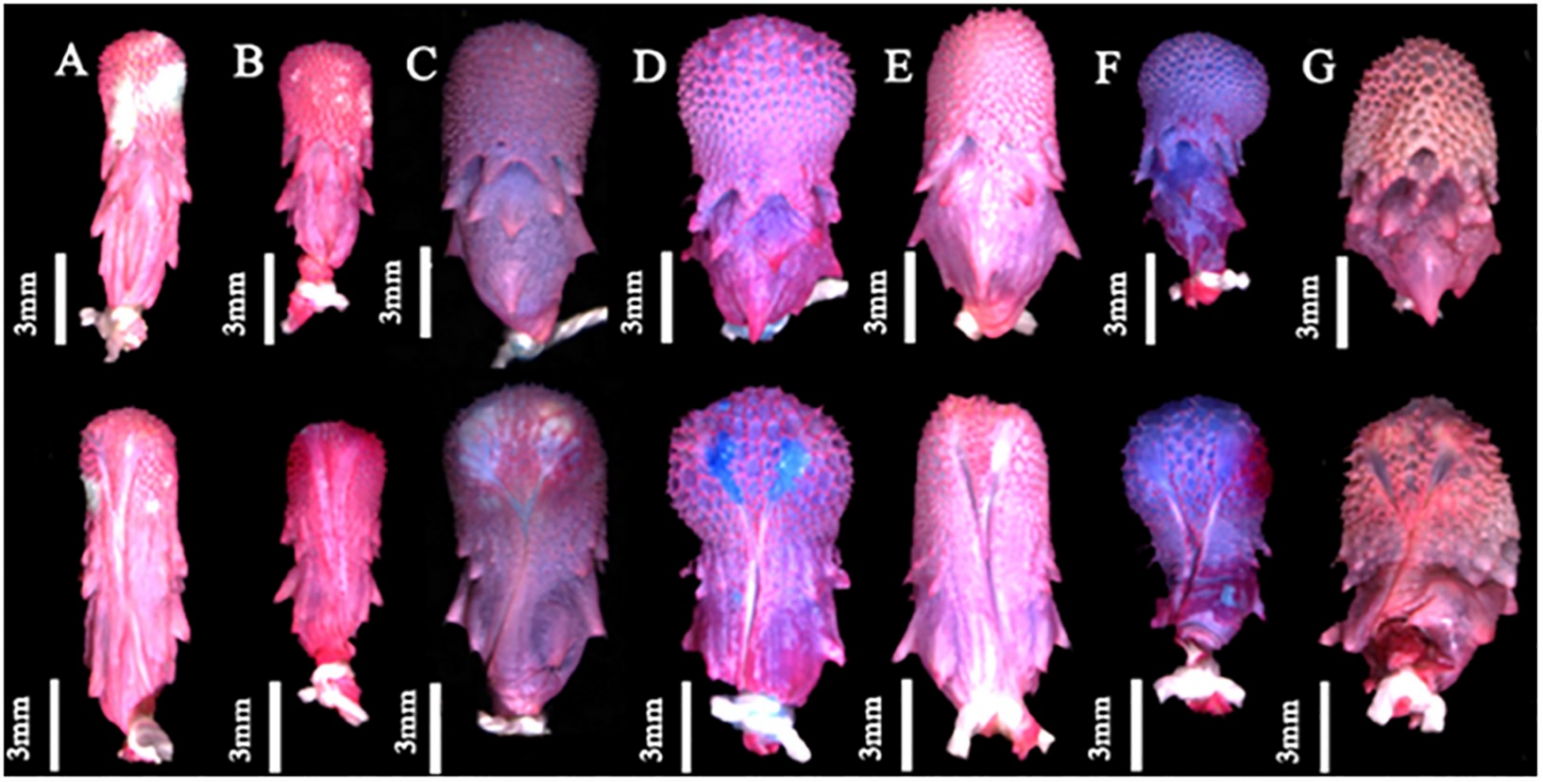
Hemipenial morphology variability in asulcate (upper) and sulcate (lower) sides of organs of *Xenopholis scalaris*: *X*. *scalaris* from municipality of Magé, state of Rio de Janeiro, Brazil (A—IVB 3552); from municipality of Jaqueira, state of Pernambuco, Brazil (B—URCA 6210); from municipality of Canavieiras, state of Bahia, Brazil (C—CZGB 1089); from locality of Campamento San Jacinto, district of Trompeteros, region of Loreto, province of Loreto, Peru (D—CORBIDI 1512); from municipality of Paranaita, state of Mato Grosso, Brazil (E—ZUEC 3443,); from locality of Campamento Bajo algodon, district of Putumayo, region of Loreto, province of Putumayo, Peru (F—CORBIDI 17429); and from locality Sierra del Divisor, district of Yaquerana, Loreto region, province Requena, Peru (G—CORBIDI 2447).

**Fig 6:**
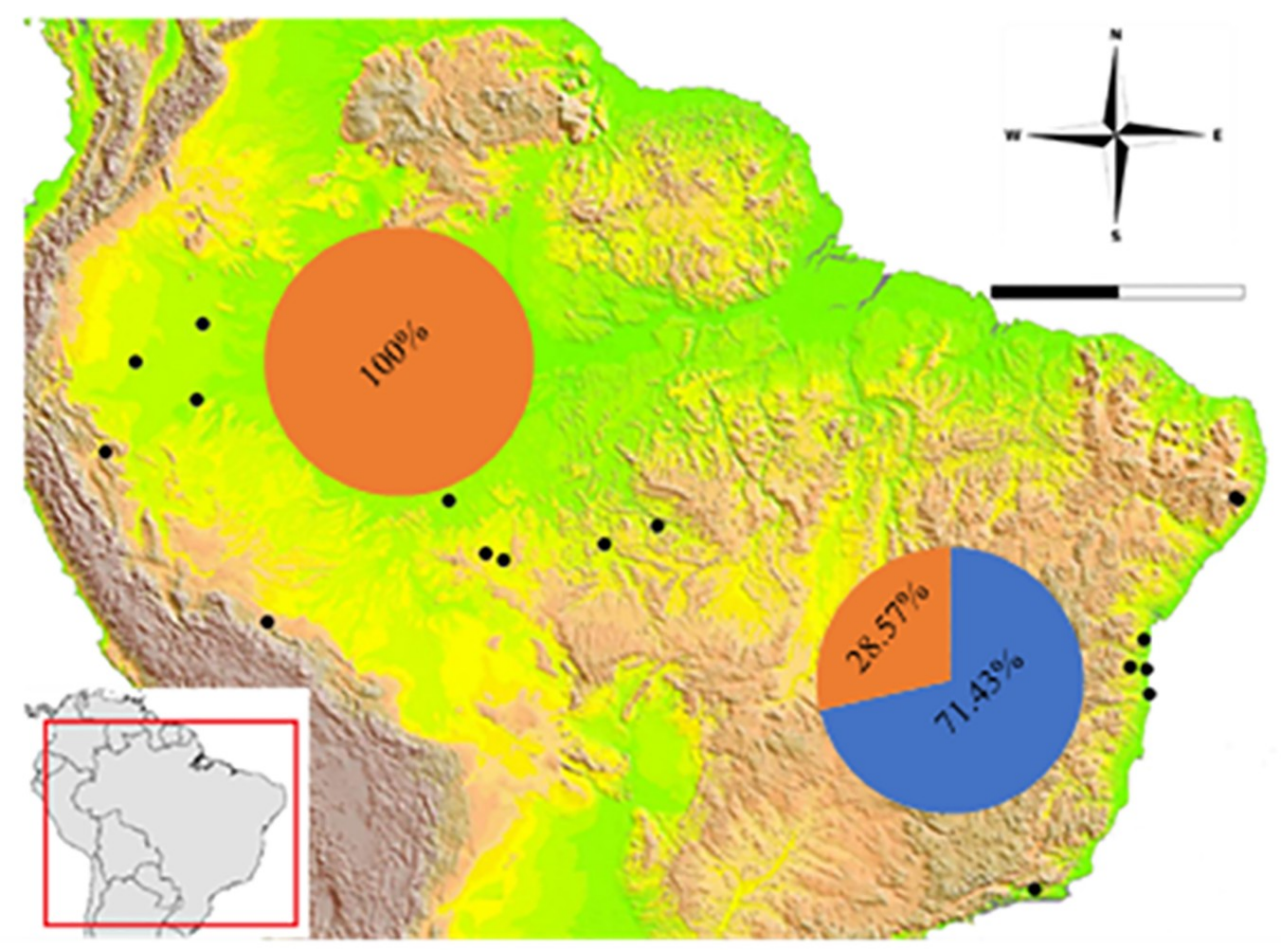
Population frequency of the hemipenes features through distribution of *Xenopholis scalaris*, considering its disjunct set of populations at Amazonia and Atlantic forest. Graphs referring to the hemipenian variability of *X*. *scalaris*. For the population of the Atlantic Forest, 71.43% presented capitulum smaller than hemipenial body (blue) and 28.57% capitulum as long as the hemipenial body (orange) (100% of Amazonian hemipenis have capitulum equivalent to hemipenial body). Digital elevation model (DEM—GTOPO30) source: U.S. Geological Survey’s EROS Data Center in Sioux Falls, South Dakota. U.S. Geological Survey’s Center for Earth Resources Observation and Science (EROS) (open source).

The analysis of hemipenial variation revealed some unique characteristics to each previously recognized species. For *Xenopholis scalaris* (Fig 5), all hemipenes for both the Atlantic Forest (N = 7) and Amazonian (N = 10) specimens are as follows: unilobed with centrolinear sulcus spermaticus bifurcation within capitulum; capitulum and hemipenial body with similar length; well defined capitular grooves on the asulcate and lateral sides of the organs, and barely defined at sulcate face of hemipenis; no ornamentation on the proximal region of the organ; hemipenial body ornamented with nearly 10 hooked spines on both faces of the organ. Among the Atlantic Forest specimens, 71.43% of the organs have capitulum smaller than hemipenial body, and 28.57% have a capitulum as long as the hemipenial body. All Amazonian specimens present capitulum equivalent to hemipenial body in length (Fig 6).

There is no clear variation on the hemipenial morphology of *Xenopholis undulatus* (N = 2) and *Xenopholis werdingorum* (N = 3). However, the scarce sample available for both species may have biased such result.

#### Niche modeling and overlapping

**Fig 7:**
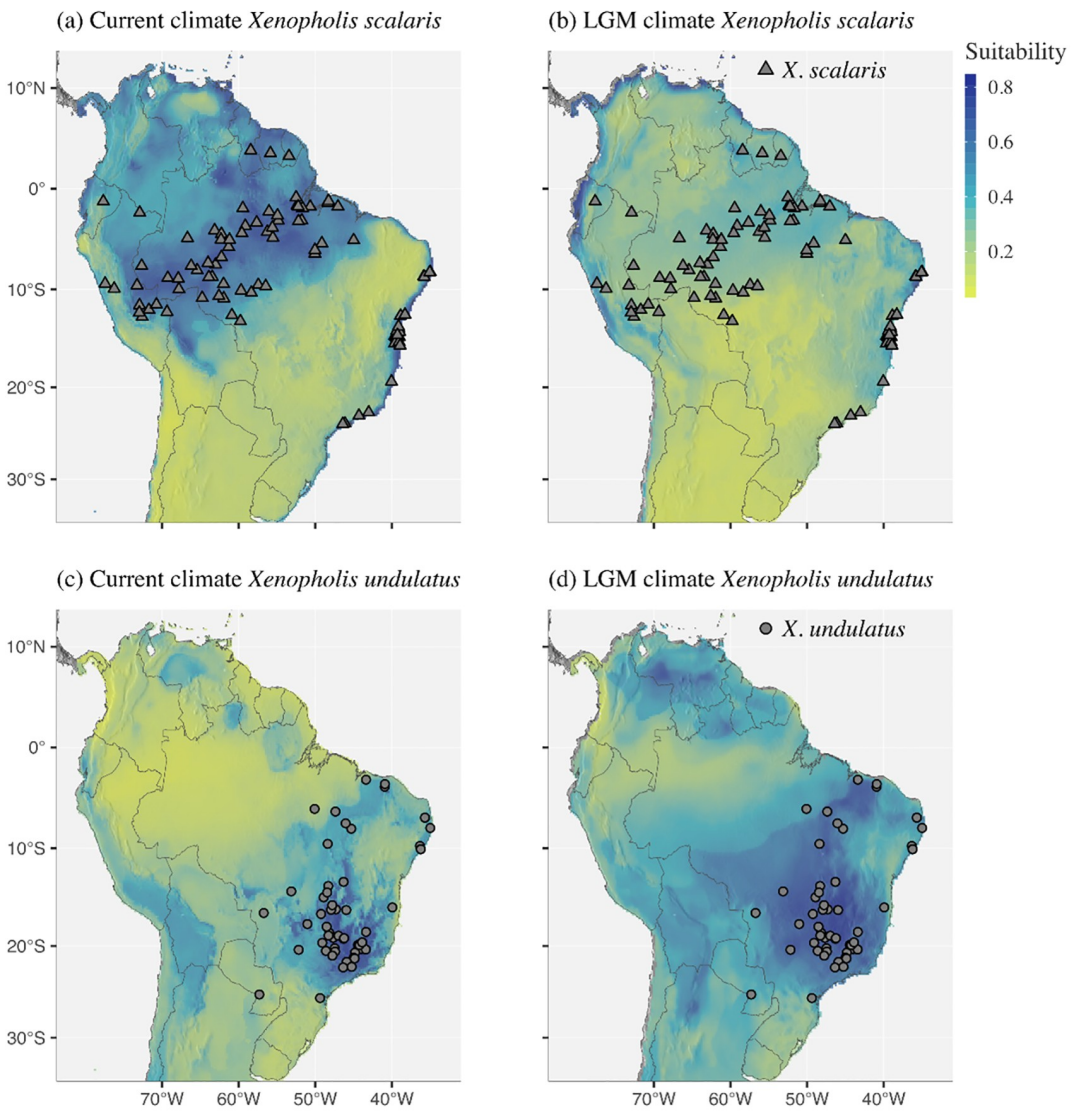
Predictions of species distribution models for: (a) Current climate and (b) Last Glacial Maximum for *Xenopholis scalaris*. (c) Current climate and (d) Last Glacial Maximum for *X*. *undulatus*. Digital elevation model source: Global Multi-resolution Terrain Elevation Data 2010 (GMTED2010). Earth Resources Observation and Science (EROS) Center (open source). DOI: 10.5066/F7J38R2N.

**Fig 8:**
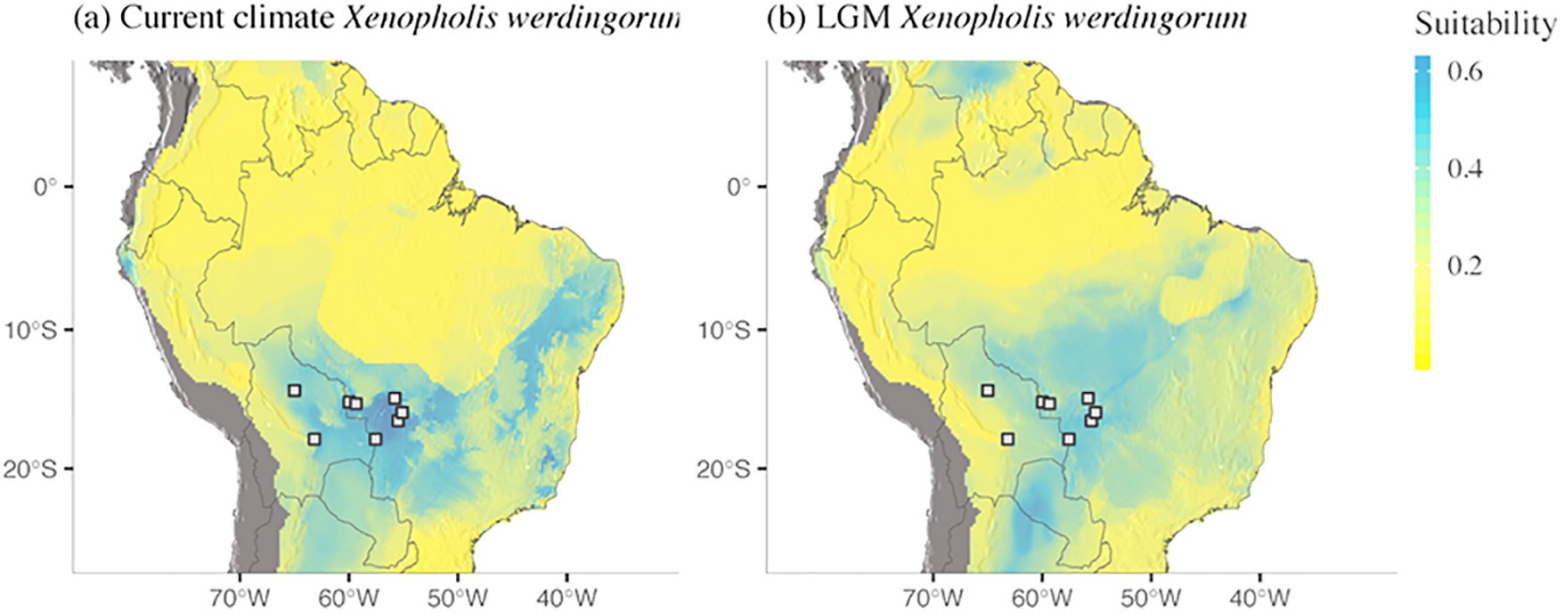
Predictions of species distribution for *Xenopholis werdingorum* using ensembling of small models. (a) Current climate. Areas with high suitability are distributed in the Pantanal basin and also beyond the known range of this species in the lowlands of Caatinga, Cerrado and Chaco. (b) Last Glacial Maximum (LGM). The model for the LGM does not differ considerably, but suitability values are generally smaller for the past conditions. Digital elevation model source: Global Multi-resolution Terrain Elevation Data 2010 (GMTED2010). Earth Resources Observation and Science (EROS) Center (open source). DOI: 10.5066/F7J38R2N.

The set of variables selected as important for each species and used in the models was unique for each species (S2 Table). All models used in the ensembling forecast presented consistent performance, with AUC values ranging from 0.76 to 0.89 (scale from 0 to 1) and TSS ranging from 0.42 to 0.68 (scale from -1 to +1, with positive values meaning better performance than random). The final model for *Xenopholis scalaris* under the current climate predicts a vast region with highly suitable environments in Amazonia and a narrow zone with suitable environments along the Brazilian Atlantic Forest (Fig 7a). The projection for *X*. *scalaris* for the LGM climate indicates that the distribution of suitable environments for this species might have been more limited back then (Fig 7b). For *X*. *undulatus*, areas with higher suitability are distributed mostly on the highlands of the Cerrado, and in intermediate values in the remaining lowlands and valleys of Cerrado and on the highlands the Caatinga (Fig 7c). Different from *X*. *scalaris*, areas with highly suitable conditions might have been more widely distributed in the LGM for *X*. *undulatus* (Fig 7d). None of the current projections for both species indicate highly suitable areas coincident with the known distribution of *X*. *werdingorum*. The ensemble of small models for *X*. *werdingorum* (AUC = 0.66–0.88, TSS = 0.09–0.15; i.e., slightly better than random), indicate highly suitable areas were predicted mostly in the Pantanal, Chaco and additional seasonally-dry tropical formations in Bolivia and Brazil (Fig 8), but not in the highlands inhabited by *X*. *undulatus*.

Niche overlap was generally low between all pairs of species (D < 0.21, Table 2), especially between *X*. *scalaris* and *X*. *werdingorum* (D = 0.08), whereas niche overlap between disjunct populations of *X*. *scalaris* in Amazonia and the Atlantic Forest was slightly higher (D = 0.24). Niche equivalence was different than the null distribution (95% confidence interval) for most pairs of species, with exception of the comparison between the populations of *X*. *scalaris* in Amazonia and the Atlantic Forest. The only comparison that indicates significantly low niche similarity was between *X*. *undulatus* and *X*. *werdingorum*.

**Table 2:** Niche overlap (Schoener’s D metric) results for each pair of species and for the for the disjunct *Xenopholis scalaris* populations from Amazonia and Atlantic forest.

Species (a − b)	D-metric	Equivalency	Similarity (a − b)	Similarity (b − a)
*X*. *scalaris—X*. *undulatus*	0.21	1.00	0.11	0.07
*X*. *scalaris—X*. *werdingorum*	0.08	0.99	0.17	0.18
*X*. *undulatus—X*. *werdingorum*	0.14	0.99	0.02	0.02
*X*. *scalaris* Amazonia—Atl. Forest	0.24	0.91	0.08	0.13

P-Values are indicated for the two distinct randomizations (1,000 randomizations) = Niche Equivalency and Niche Similarity. Most results indicate significantly low values of niche equivalence (outside the 95% of the null distribution values).

#### Taxonomic decision

Based on the results obtained through the congruence of our quantitative and qualitative phenotypic analyses evaluated here in combination with niche modeling and niche overlapping, it was not possible to distinguish among the Amazonian and Atlantic Forest populations of *Xenopholis scalaris* as independent taxonomic units. In contrast, the currently recognized species were widely discriminated considering all sources of phenotypic characters studied in combination with very distinct niche ecologies for each taxa. In this way, we choose to maintain the current taxonomic arrangement for the genus *Xenopholis*, improving the diagnosis of each previously recognized species.

#### *Xenopholis scalaris* (Wucherer, 1861)

*Elapomorphus scalaris* Wucherer, Proc. Zool. Soc. of London 1861:325. (two syntype from municipalities of Canavieiras 15°39’1”S 38°57’42”W and Mata de São João 12°31’50”S 38°17’59”W, state of Bahia, Brazil).

*Xenopholis braconnieri* Peters, Monatsberichte der Koniglichen Preussische Akademie des Wissenschaften zu Berlin 1869:441. (unknown provenance).

*Gerrhosteus prosopis* Cope, Proceedings of the Academy of Natural Sciences of Philadelphia 1874:71. (two syntype collected by Professor James Orton at Nauta on the Peruvian Amazon).

*Sympeltophis ungalioides* Werner, 1925:52 Sitzb. Nath. Naturwiss. Akad. Wiss. Wien 1, 134:52. (from Central Brazil).

*Comparative diagnosis*. *Xenopholis scalaris* can be distinguished from all congeners by the following characters combination: (1) dorsum of head from red to reddish-brown in life, and light brown or pale brown after preservation (vs. black in *X*. *undulatus* and *X*. *werdingorum* in life and after preservation); (2) dorsal ground color of body red, reddish-brown to orange in life and light or pale brown after preservation, with black alternated paravertebral blotches, sometimes connected forming conspicuous cross-bands (vs. dorsal ground color covered with conspicuous black, with a broad and irregular vertebral stripe in *X*. *undulatus*, and dorsum black with three paraventral scale rows, orange in life and pale brown after preservation in *X*. *werdingorum*); (3) dorsal scales rows 17/17/17 (vs. 19/19/17 in *X*. *undulatus* and *X*. *werdingorum*); (4) ventral scales in males 126–169, 128–175 in females (vs. 160–190 in males of *X*. *undulatus* and 181–195 of *X*. *werdingorum*, 168–196 in females of *X*. *undulatus* and 180–196 of *X*. *werdingorum*); (5) subcaudal scales 28–45 in males, 27–42 females (vs. 36–55 in males of *X*. *undulatus* and 46–54 of *X*. *werdingorum*; and 33–60 in females of *X*. *undulatus* and 38–48 of *X*. *werdingorum*); (6) postocular scale single (vs. two postoculars in *X*. *undulatus* and *X*. *werdingorum*); (7) hemipenis unilobed with bifurcated sulcus spermaticus (vs. unilobed usually with single sulcus spermaticus in *X*. *undulatus* and bilobed organ in *X*. *werdingorum*); (8) hemipenis strongly capitulated on the sulcate side (vs. slightly capitulated in *X*. *undulatus* and not capitulated in *X*. *werdingorum*); (9) capitulum ornamented with spinulate calyces (vs. papillate on distal portion of capitulum in *X*. *undulatus* and entirely papillate in *X*. *werdingorum*); (10) hemipenial body ornamented with hooked spines and longitudinal plicae (vs. hemipenial body ornamented with hooked spines and dispersed papillae in *X*. *undulatus* and *X*. *werdingorum*); (11) pupil red (vs. brown in *X*. *undulatus* and *X*. *werdingorum*); (12) neural spine of vertebrae without septum perpendicular to longitudinal axis of body (vs. presence of a narrow longitudinal septum in *X*. *undulatus* and *X*. *werdingorum*); (13) vomerian process of premaxillae contacting mesolateral portions of vomers (vs. overlapping vomers in *X*. *undulatus* and *X*. *werdingorum*); (14) nasal process present (vs. absent in *X*. *undulatus*); (15) pair of nasals about the same length of frontals (vs. smaller than frontals in *X*. *undulatus* and *X*. *werdingorum*); (16) dorsal crests of parietal not contacting each other (vs. contacting each other in *X*. *werdingorum*); (17) no contact between frontals and postorbitals (vs. contact present in *X*. *undulatus* and *X*. *werdingorum*); (18) contact between supratemporals and supraoccipital present (vs. absent in *X*. *undulatus*); (19) seven palatine teeth (vs. 10 in *X*. *undulatus* and *X*. *werdingorum*); (20) 28 teeth in the pterygoids (vs. 14 in *X*. *undulatus* and 23 in *X*. *werdingorum*).

**Fig 9:**
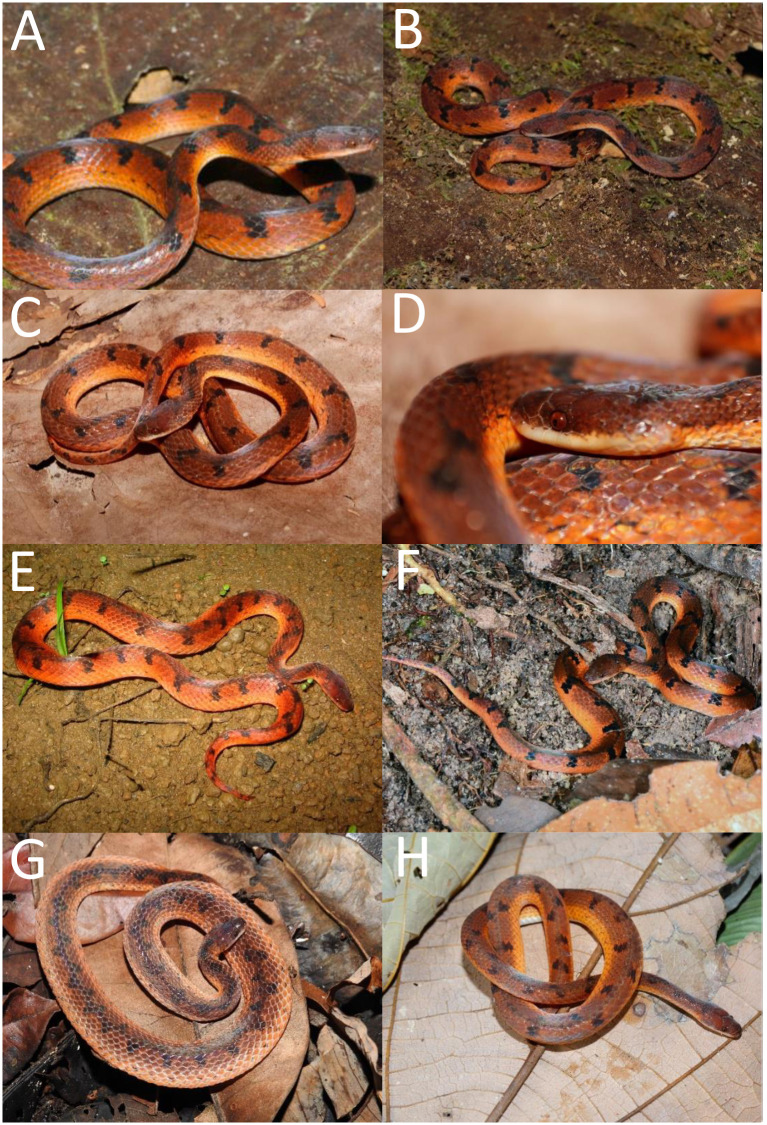
Color variability of the *Xenopholis scalaris* in life. A—tributary between Madeira and Purus Rivers, state of Amazonas, Brazil; B—D municipality of Juara, state of Mato Grosso, Brazil; E—municipality of Una, state of Bahia, Brazil; F—Itapuã do Oeste, state of Rondônia; G—Murici, state of Alagoas; H—Assis Brasil, state of Acre. Photos by V. Carvalho (A); T. Rodrigues (B–D); M. A. Freitas (E); D. Meneghelli (F); M. A. Freitas (G–H).

*Color pattern in life (*Fig 9*)*. Dorsum of head and body reddish-brown; dorsal ground color of the body reddish-brown along 6th to 12th scale rows, with black alternated paravertebral blotches, sometimes connected and forming conspicuous cross-bands (one to two scales long); paravertebral blotches or bands generally extending three or four scale rows in the vertebral region on each side of body; first five scale rows usually uniformly orange; sometimes paraventral rows covered with few black marks (dots or spots) on the limit of lighter paraventral region along fifth scale row; supralabials mostly creamish white with little invasion of red pigmentation on its dorsal edges; ventral surface of body uniformly creamish white to creamish yellow. Iris red.

*Color variability observed in preserved specimens (*S1 Fig*)*. The color pattern after preservation is very similar to coloration in life, only changing to fading red, orange, and yellow pigments. The orange and red pigments become pale brown and brown, respectively; while yellow and creamish yellow become cream.

*Quantitative variability for secondarily dimorphic characters*. Number of ventral scales 126–169 (mean = 135.25, SD = 6.11, N = 115) in males, 128–175 (mean = 139.33, SD = 7.33, N = 131) in females; number of subcaudal scales 28–45 (mean = 35.90, SD = 3.73, N = 115) in males, 27–42, (mean = 32.09, SD = 2.46, N = 131) in females; and number of preoculars in males 1–2 (mean = 1.09, SD = 0.28, N = 104), 1–2 (mean = 1.04, SD = 0.17, N = 128) in females. We refer to Table 3 for variables with no sexual dimorphism.

**Table 3:** Selected variables synthesizing the meristic and morphometric variation of *Xenopholis scalaris*.

	Min	Max		SD	-95%	95%	N
SVL (mm)	110	395	243.30	42.27	238.35	248.51	247
CL (mm)	15	75	48.36	9.80	47.13	49.59	247
TL (mm)	125	433	292.07	50.43	285.75	298.39	247
Distance nostril (mm)	1.10	2.72	1.92	0.31	1.88	1.96	229
Eye circumference (mm)	0.85	2.27	1.22	0.16	1.20	1.24	230
Dist. nostril-eye (mm)	1.14	3.73	2.37	0.41	2.32	2.43	229
Dist. rostral-eye (mm)	2.00	4.35	3.35	0.47	3.29	3.42	229
Dist. eye (mm)	2.15	4.47	3.20	0.36	3.15	3.25	230
Head length (mm)	6.75	14.70	10.87	1.35	10.69	11.05	230
Head width (mm)	3.30	7.49	5.38	0.74	5.29	5.48	230
Head height (mm)	2.27	4.67	3.45	0.45	3.39	3.57	229
Dorsal I	17	17	17	0	-	-	247
Dorsal II	17	17	17	0	-	-	247
Dorsal III	17	17	17	0	-	-	247
First temporal	1	1	1	0	-	-	246
Second temporal	1	3	2	0.14	1.96	2.00	246
third temporal	2	4	3	0.30	2.86	2.94	246
Supralabial	7	8	7.99	0.08	8	8	246
Larger supralabial	6	7	6.99	0.10	6.97	7.00	246
Geniais	4	4	4	0	-	-	246
Infralabial	8	9	8.99	0.09	8.98	9.00	244
1° supralabial-eye	3	4	3.99	0.09	3.97	4.00	232
2° supralabial-eye	4	5	4.99	0.09	4.97	5.00	232
Postocular	1	3	1.94	0.24	1.91	1.97	231
IL cont. 1° ment.	1	4	1	0	-	-	231
IL cont. 2° ment.	4	5	4.01	0.11	3.99	4.02	231
Prefrontal	1	3	1.01	0.18	0.99	1.04	232
Maxillary teeth	10	12	11.85	0.36	11.81	11.90	225
Number of spots	24	41	33.08	2.97	32.70	33.45	246

Abbreviations are as follows: CL = caudal length; SVL = snout-vent length; TL = total length; IL cont. 1°/2° ment. = Infralabial contact with the first/second mentonian; Max = maximum; Min = minimum; N = sample size; SD = standard deviation; -95% = lower limit of the confidence interval; + 95% = upper limit of the confidence interval.

**Fig 10:**
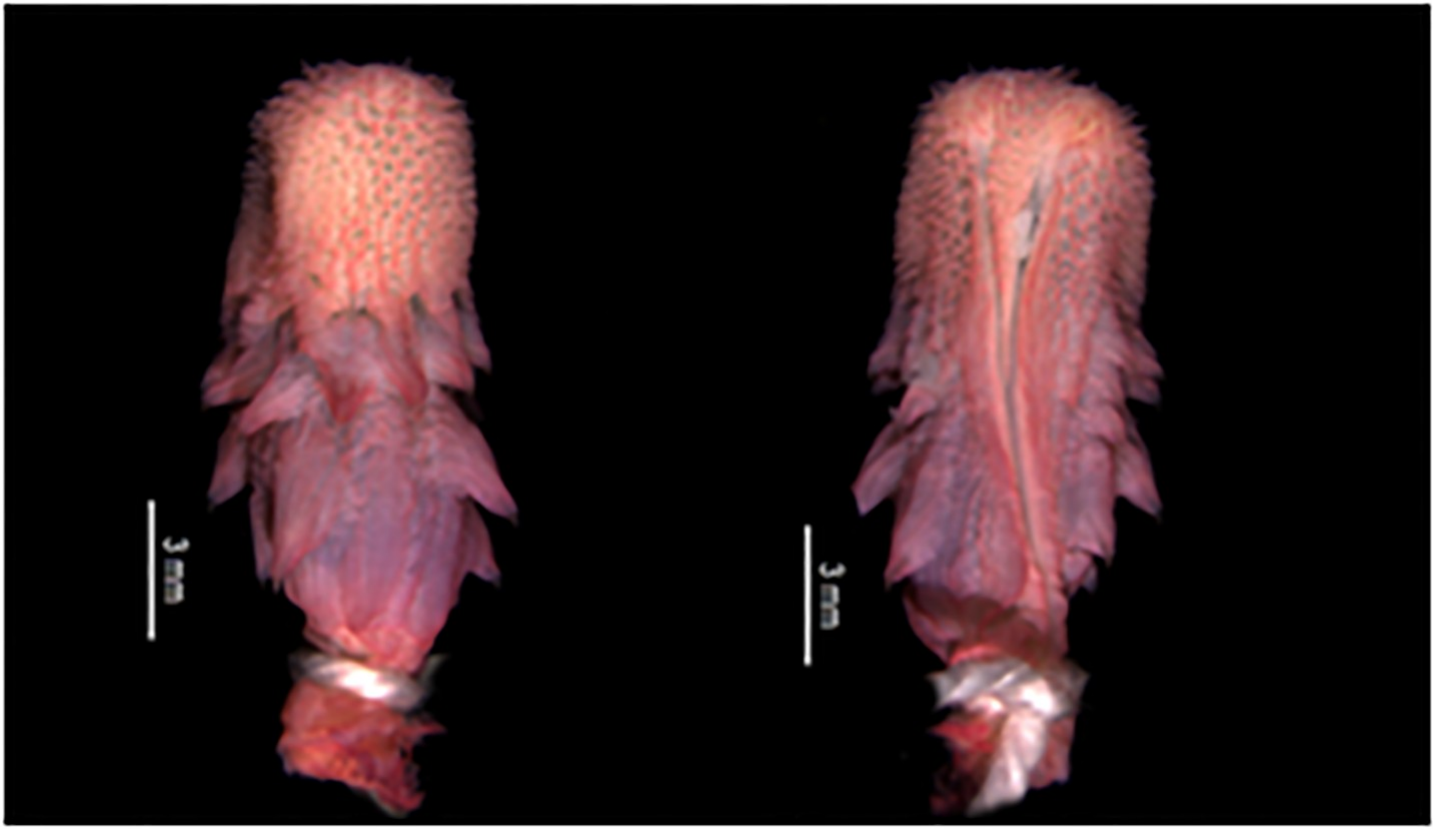
Asulcate (A) and sulcate (B) side of the hemipenis of *Xenopholis scalaris* from municipality of Almadina, state of Bahia, Brazil (CZGB 13474).

*Hemipenial morphology (*Fig 10*)*. Fully everted and maximally expanded hemipenes rendered a unilobed, unicalyculate and semicapitate organ; capitulum similar or barely slender than hemipenial body; capitular crotch strongly developed on the asulcate side and nearly indistinct at sulcate face of hemipenis; capitulum clavate or almost attenuated and similar or shorter than hemipenial body; capitulum uniformly covered by spinulate calyces; basal region of capitulum on the asulcate and lateral faces with hooked spines entering hemipenial body through capitular crotch; hemipenial body elliptical and scattered with large hooked spines; hemipenial body usually covered with three rows of hooked spines (5/5/2), almost transversally arranged from the capitular groove to proximal region of hemipenial body; sulcate and lateral faces of hemipenis with six/seven hooked spine on each side of the sulcus spermaticus; hemipenial body with longitudinal plicae among hooked spines; larger spines generally located laterally below sulcus spermaticus bifurcation; sulcus bifurcates for about half of organ within capitulum, with each branch centrolinearlly oriented and running to the distal region of capitulum, but not reaching its apex; sulcus spermaticus margins expanded after sulcus bifurcation and not bordered by spinules; basal naked pocket absent or indistinct; most basal region of hemipenis without spinules and with longitudinal plicae.

**Fig 11:**
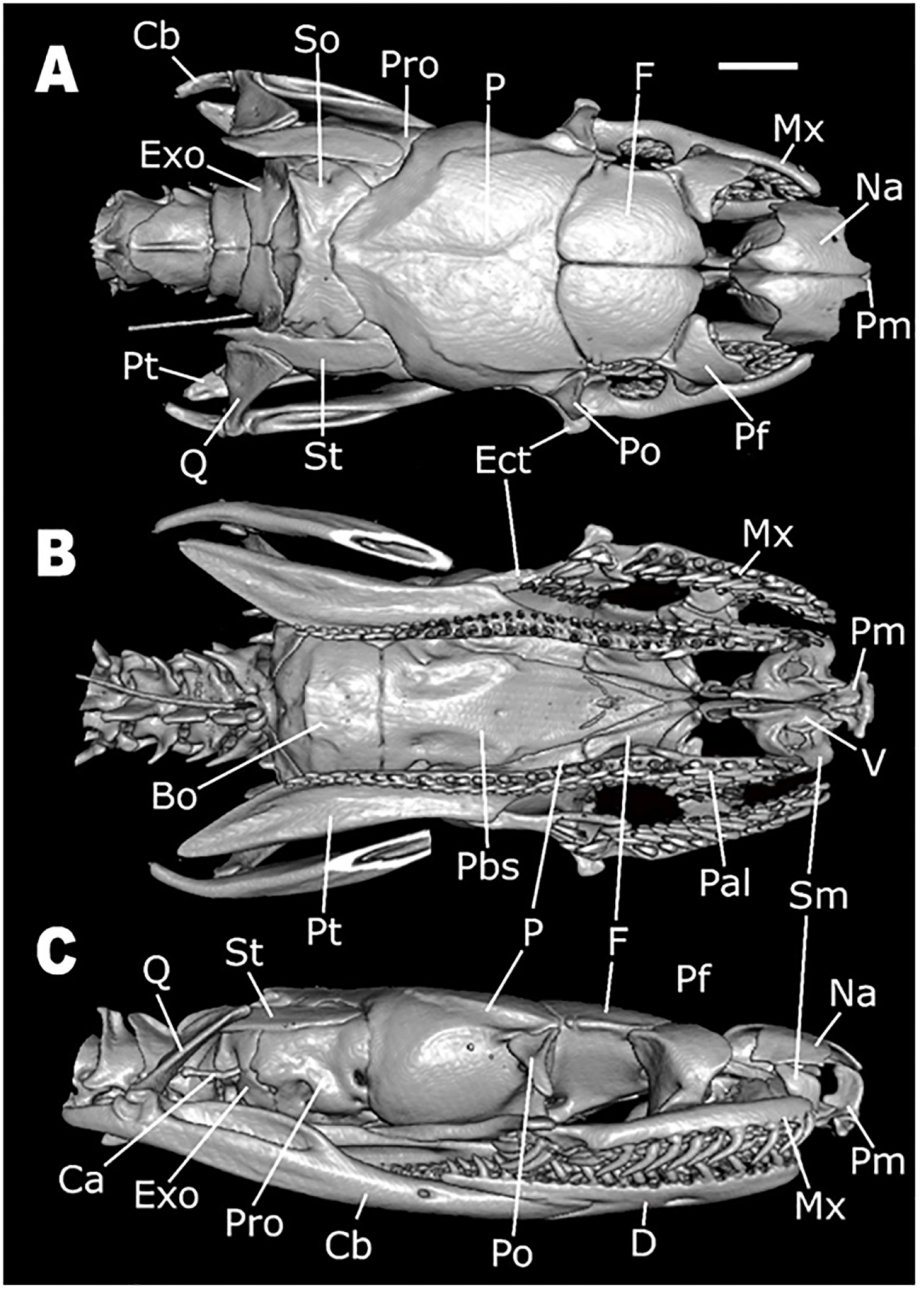
Dorsal (A), ventral (B), and lateral (C) views of the skull of *Xenopholis scalaris* (MNRJ 17070) from Cabo de Santo Agostinho, state of Pernambuco. Abbreviations are as follow: Cb = compound bone; So = supraoccipital; Pro = prootic; P = parietal; F = frontal; Mx = maxilla; Na = nasal; Pm = premaxilla; Pf = prefrontal; Po = postorbital; Ect = ectopterygoid; St = supratemporal; Q = quadrate; Pt = pterygoid; Exo = exoccipital; V = vomer; Sm = septomaxilla; Pal = palatine; Pbs = parabasisphenoid; Bo = basioccipital; Ca = *columella auris*; and D = dentary.

**Fig 12:**
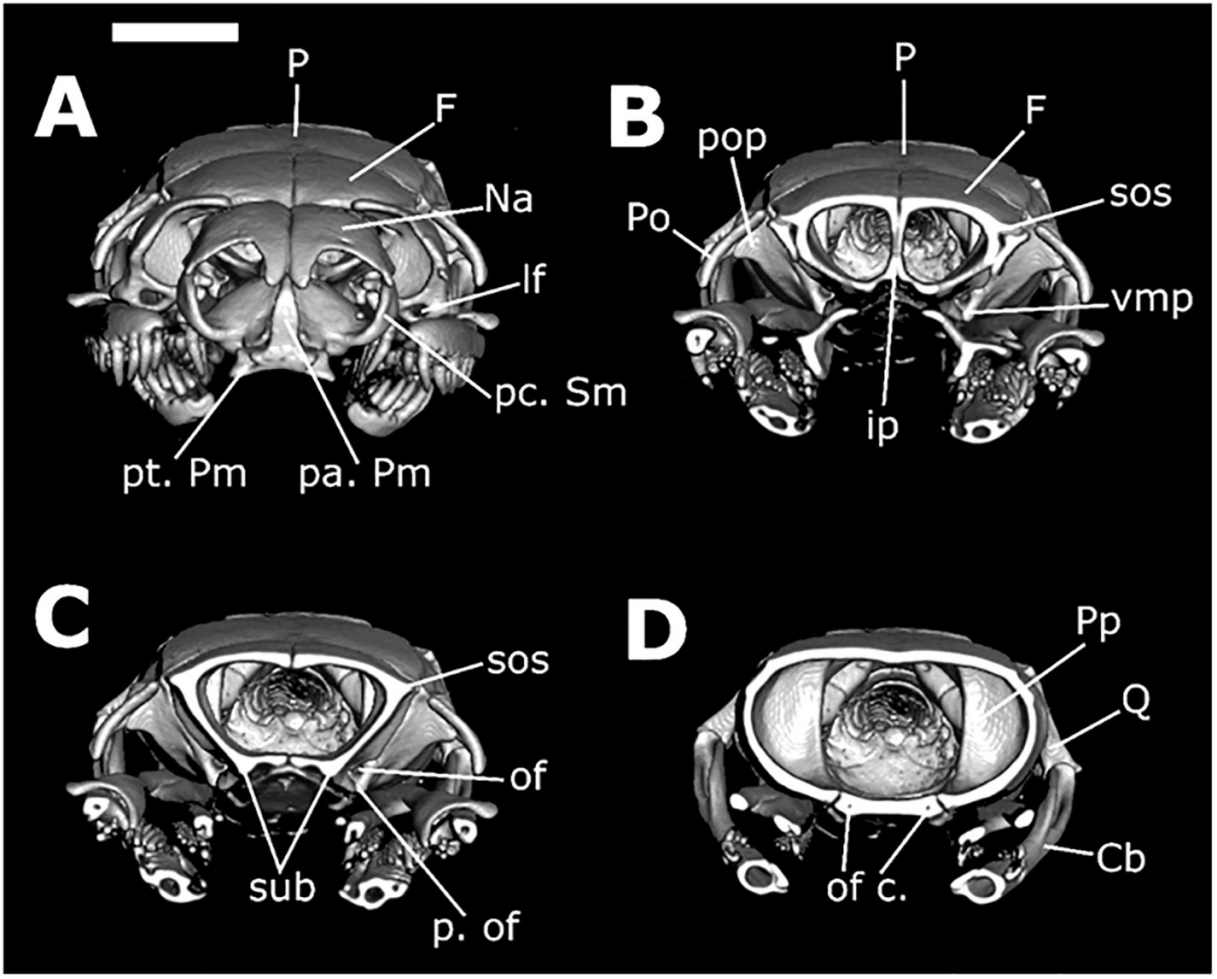
Three-dimensional cutaway views along the transverse axis of *Xenopholis scalaris* (MNRJ 17070), from the anterior edge of the skull. Abbreviations are as follow: pt. Pm = transverse process of premaxilla; pa. Pm = ascendant process of premaxilla; pc. Sm = conchal process of septomaxilla; lf = lacrimal foramen of prefrontal; Po = postorbital; P = parietal; sos = frontal supraorbital shelf; ip = interolfactory pillar of frontal; of = optic foramen; p. of = parietal process of optic foramen; sub = subolfactory process of frontal; of c. = optic foramen canal of parabasisphenoid; Cb = compound bone; Q = quadrate; Pp = parietal pillar.

**Fig 13:**
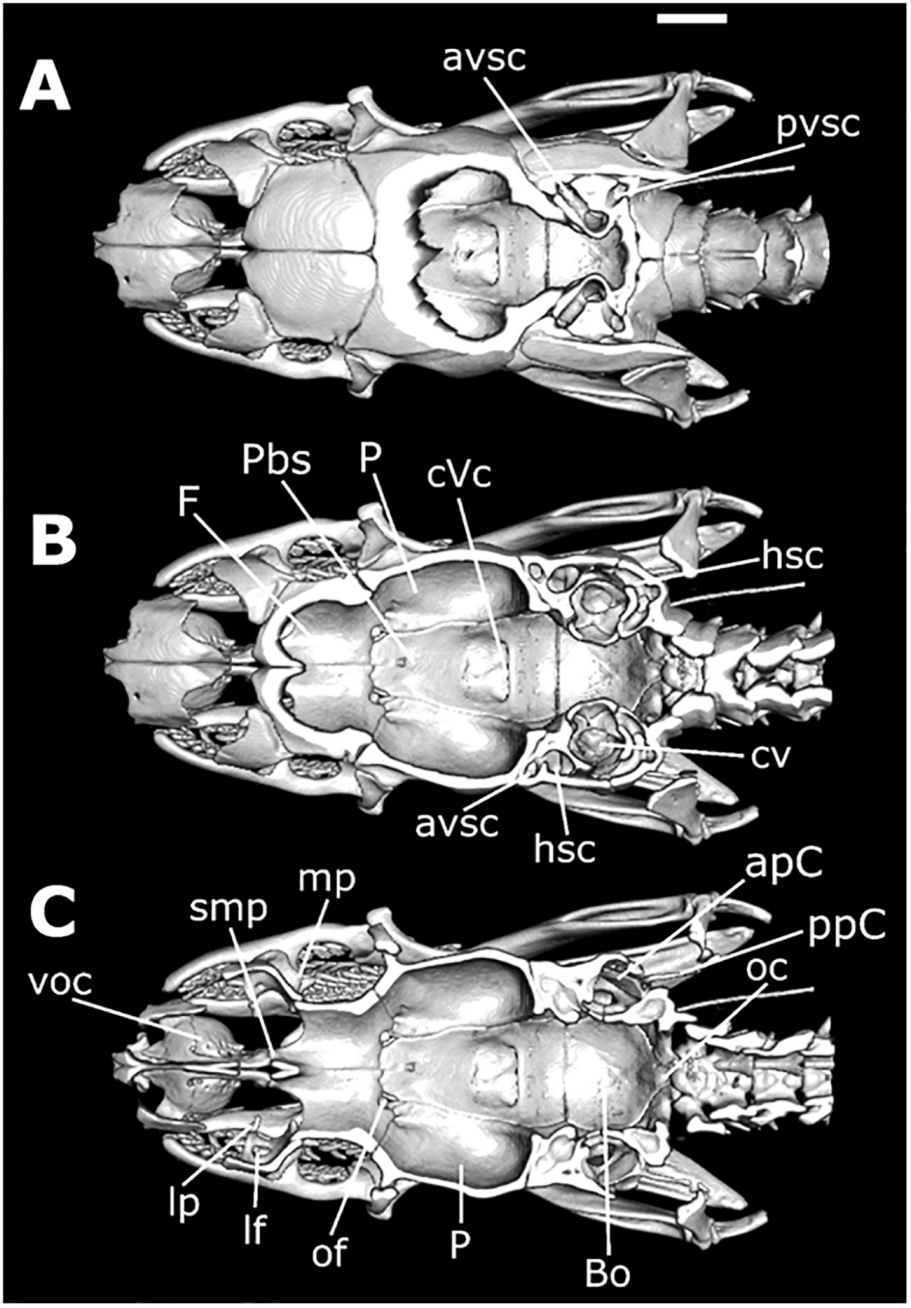
Three-dimensional cutaway views along the longitudinal axis of *Xenopholis scalaris* (MNRJ 17070), from the dorsal edge of the skull. Abbreviations are as follow: avsc = anterior vertical semicircular canal; pvsc = posterior vertical semicircular canal; cVc = crest of the Vidian foramina; hsc = horizontal semicircular process; cv = cavum vestibuli; mp = mesomedial process of prefrontal; smp = septomaxilar process of frontal; voc = vomeronasal organ capsule; lp = lacrimal process of prefrontal; lf = lacrimal foramen of prefrontal; of = optic foramen; P = parietal; Bo = basioccipital; oc = occipital condyle.

**Fig 14:**
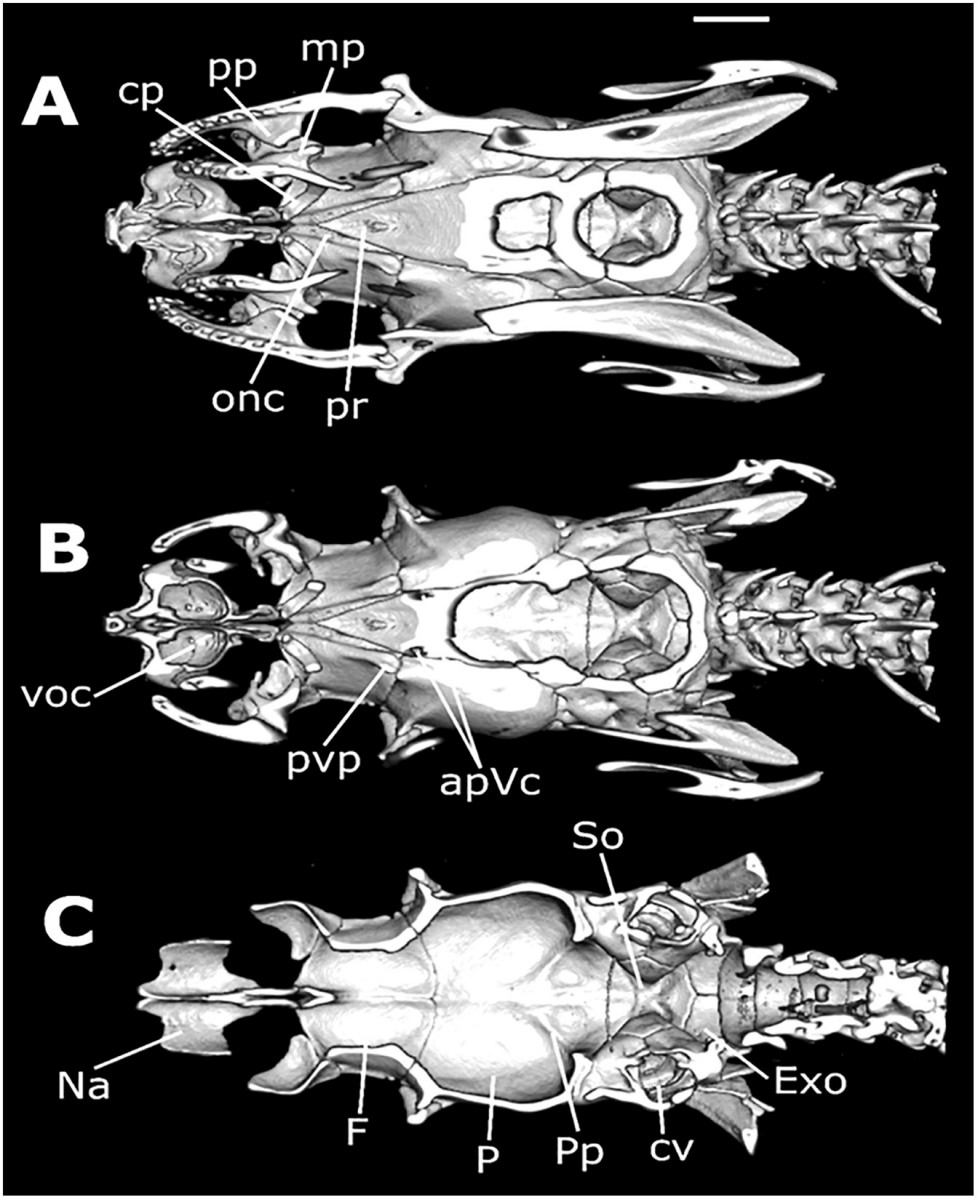
Three-dimensional cutaway views along the longitudinal axis of *Xenopholis scalaris* (MNRJ 17070), from the ventral edge of the skull. Abbreviations are as follow: cp = choanal process of palatine; pp = palatine process of maxilla; mp = maxillary process of palatine; onc = optic nerve canal; pr = parasphenoid rostrum; voc = vomeronasal organ capsule; pvp = posteroventral process of frontal; apVc = anterior opening of Vidian canal; So = supraoccipital; Exo = exoccipital; cv = cavum vestibuli; Pp = parietal pillar; P = parietal; F = frontal; Na = nasal.

*Skull morphology (*Figs 11–14*)*. SNOUT COMPLEX: *Premaxilla*: delimits the skull anteriorly, contacting nasals dorsoposteriorly (Fig 11a) and septomaxillae in its posteromesial portion (Fig 11c); narrow and ventrally inclined transverse processes (Fig 12a), posteriorly oblique, not contacting maxillae (Fig 11); ascendant process with a pair of lateral projections on its base (Fig 12a); base of ascendant process wider than its tapered dorsal edge, which is inserted between pair of nasals (Fig 12a); vomerian processes short and divergent, contacting anteromedial portion of vomers (Fig 11b); nasal process present (Fig 11c). *Septomaxillae*: located dorsally to the vomers, ventral to the nasals and posteriorly to the premaxilla (Fig 11); together with the vomers, forms the vomeronasal organs capsule (voc—Figs 13 and 14); conchal process with tapered edge posteriorly turned, not overlapping with the anterior portion of the maxilla or with transverse process of the premaxilla (Fig 11); conchal process in contact with the mesolateral portion of nasals (Fig 12a); reduced contact with the nasals, forming a large orifice bordered by the premaxilla, nasals and the septomaxillae (Fig 11c); posterior portion in contact with the septomaxilar process of the frontal bone (Fig 13c). *Vomers*: located in the anteroventral portion of the skull, posteroventrally to the premaxilla (Fig 11b); anterior process laterally to the vomerian process of premaxilla, contacting it (Fig 11b); mesolateral projection not overlapped by the palatines; posterior process with small foramen in its ventral portion and vertical lamina concave. *Nasals*: located in the dorsal surface of the skull, posteriorly to the premaxilla and anteriorly to the frontals, not contacting it (Fig 11a); large in dorsal view, about the same extension as the frontals; mesial portion with wide lateral process that curves ventrally, with straight edge contacting conchal process of septomaxilla (Fig 11c); ascendant process of premaxilla inserted between the pair of nasals, in its anterior portion (Fig 11a); short frontal process, not contacting the frontal; in lateral view, recess on the anteroventral portion, forming an orifice bordered by the premaxilla, septomaxillae and the nasals (Fig 11c).

BRAINCASE: *Frontals*: located in the dorsal surface of the skull, posteriorly to the nasals and anteriorly to the parietal (Fig 11); contacts the prefrontals in the anterolateral region; inter-olfactory pillar located on its anterior portion (Fig 12b), visible in frontal view, with small septomaxillar processes ventrally contacting the septomaxilla; frontal with about the same extension of nasals and half the extension of the parietal in dorsal view (Fig 11a), not contacting the nasals; anterior margins straight and oblique with respect to lateromedial axis; posterior margins slightly concave and oblique regarding the lateromedial axis (Fig 11a); lateral margins straight, with anterior and posterior portions about the same width; prefrontal process absent; suture with prefrontal oblique; in dorsal view, pair of frontals wider than longer, not contacting postorbital; in lateral view, small posteroventral process, delimiting ventral surface of the optical foramen, which is small, with less than the interorbital septum height, totally inserted in the frontals, with parietal only bordering its posterior margin (Fig 11c); in frontal view, frontal subolfactory process enclosing the optic nerve canal (sub—Fig 12c); frontal supraorbital shelf present (sos—Fig 12b and 12c). *Parietal*: located posteriorly to frontals, contacting it anteriorly (Fig 11a); contacts supraoccipital posteriorly, prootic posterolaterally (Fig 11a), and parabasisphenoid ventrolaterally (Figs 13c, 14a and 14b); anterolaterally portion contacts postorbital in dorsal view (Fig 11a); parietal does not contact supratemporals or close the braincase cavity ventrally, which is enclosed by parabasisphenoid (Fig 11b); subtriangular shape in dorsal view; sutures with frontal convex, given anterior margin a convex aspect with a small projection on the mesial portion (where the pair of frontals meet each other) (Fig 11a); small postorbital process; posterior margin convergent with parietal-exoccipital suture convex; dorsolateral crests slightly developed, convergent, not contacting each other, originating in the most anterolateral point of parietal and converging to suture with exoccipital at its medial region; in frontal view, two small processes (one on each side) on the ventromedial portion, which form the border of orbital foramina, and a pair of postorbital processes (Fig 12b). *Supraoccipital*: located in dorsal surface of skull, contacting parietal anteriorly, exoccipitals posteriorly, prootic anterolaterally, and laterally supratemporals (Fig 11a); anterior margin convex in dorsal view; dorsolateral crests of parietal continue over supraoccipital, becoming a transversal crests on the posterior region of the bone, which corresponds to dorsal surface of semicircular canal; longitudinal crest originates in the medial portion of transversal crests (Fig 11a); transversal crests slightly oblique relative to lateromedial axis; both lateral portions of supraoccipital form cavities inside, which begin at about the middle line of the bone; two dorsal canals correspond to anterior and posterior vertical semicircular canals (avsc and pvsc, Fig 13) and a ventral canal belongs to cavum vestibuli (cv, Fig 13), connecting to prootic and exoccipitals—semicircular canals and cavum vestibuli forms the ear capsule; its dorsal surface bears two pairs of small foramina on the mesolateral portion. *Exoccipitals*: irregular in shape, delimiting dorsoposterior portion of the skull (Fig 11); contacts supraoccipital anteriorly and atlas posteriorly (Fig 11a); its posteroventral portion forms, with basioccipital, the occipital condyle (oc, Fig 13c), located on the ventral margin of foramen Magnum; contacts prootic anterolaterally and basioccipital ventrally (Fig 11); foramen oval located in its anteromesial portion, in lateral view—a cavity where the *columella auris* is inserted (Fig 11c); in the suture between exoccipitals and prootic there is a continuity of the foramen; other foramina are present ventral to the foramen oval; bears part of the posterior vertical semicircular canal and horizontal semicircular canal (Fig 13b), which have continuity in the supraoccipital and prootic; posterior margin straight, slightly oblique to lateromedial axis (divergent) (Fig 11a); transversal crests of supraoccipital continues at lateral portion of exoccipitals, where supratemporal relies (Fig 11); sutures with basioccipital straight. *Basioccipital*: located in the ventral portion of the skull, delimiting it ventroposteriorlly (Fig 11b); anteriorly delimited by parabasisphenoid, with suture straight, and posteriorly delimited by atlas; posterior edge forming the main portion of occipital condyle, on the margin of foramen Magnum (oc, Fig 13c); anterolateral portions contact prootic and posterolateral portions contact exoccipitals, with both sutures straights and obliques to the anteroposterior axis; shape nearly pentagonal; two small dentigerous processes, forming a slightly developed crest on its mesial portion (Fig 11b); mesolateral processes absent. *Parabasisphenoid*: composed by fusion of basisphenoid with parasphenoid; elongated triangular bone in ventral view, with anterior tip tapered (Fig 11b); located ventrally on the braincase, contacts medial portions of frontals anteriorly, basioccipital posteriorly, prootics posterolaterally, and parietal laterally; in ventral view, a pair of small foramina pierce the bone close to its posterolateral margin, corresponding to posterior opening of the Vidian canal; in dorsal view (from the inside of the endocast) there is a well delimited crest forming a cavity in which the posterior foramina of the Vidian channel opens (cVc, Fig 13b); anterior openings of the Vidian canal located on the suture with parietal, in its mesial portion (apVc, Fig 14b); in dorsal view, anterior portion of bone, the parasphenoid rostrum (pr, Fig 14a), overlapped by frontals (Fig 13b); in ventral view, edges of the parasphenoid rostrum and subolfactory processes of frontals borders the optic nerve canal (onc, Fig 14a), which is totally enclosed by the parabasisphenoid after the parasphenoid rostrum. *Prootics*: located lateroposteriorlly in the braincase (Fig 11); contacts parietal anteriorly and anterodorsally, supraoccipital posterodorsally, exoccipital posteriorly, basioccipital posteroventrally, and parabasisphenoid anteroventrally; most of the dorsal face overlapped by supratemporal (Fig 11a); in lateral view, two large foramina present, being the foramen for maxillary branch of trigeminal and the foramen for mandibular branch of trigeminal (Fig 11c); both foramina open in the interior of the braincase, and they are apart from each other by the laterosphenoid; there is a foramen, connected to foramen oval, situated in its posterior margin (foramen oval). However, the *columella auris* restricted to above the exoccipital; in dorsal view, longitudinal crest absent; there are other small foramina ventral to the maxillary and mandibular branches of trigeminal foramina. *Prefrontals*: irregular and located anterolaterally to frontals, forming anterior limit of the orbit (Fig 11); ventrally, contacts palatine process of maxilla and maxillary process of palatine; in lateral view, anterior portion with a convex projection and posterior portion concave; ventral portion narrow and dorsal portion broader (Fig 11c); lateral foramen absent; prefrontal-frontal suture oblique; lacrimal foramen visible in frontal view (lf, Figs 12a and 13c), on its ventral region, with well-developed lacrimal process (lp, Fig 13c); mesomedial process well developed (mp, Fig 13c) and posteroventral process slightly developed. *Postorbitals*: located anterolaterally to parietal, contacting only this bone (Fig 11); forms posterior limit of orbit; subtriangular shaped, with dorsal edge straight and ventral edge tapered; anterior margin slightly concave and posterior straight (Fig 11c).

PALATOMAXILLARY ARCH: *Maxillae*: located on the anterolateral portion of the skull (Fig 11); contacts ventral region of prefrontal in its mesomedial portion, through the palatine process, and the ectopterygoid in its posterior portion; does not contact premaxilla, postorbital, and palatine; arched shaped, with lateral lamina convex and medial lamina concave; bears 15 posteriorly curved prediastemal teeth of about the same size, and two postdiastemal grooved teeth, about the same size of prediastemal ones; diastema with size equivalent to one tooth socket; palatine process located on the medial face of the bone, from teeth 9–12^th^, with tapered edge posteriorly curved (pp, Fig 14a); posterior portion of maxilla wider. *Palatines*: located on the medial portion of the palatomaxilar arch, in the ventral face of the braincase (Fig 11b); contacts pterygoid on its posterior portion and prefrontal through the maxillary process; bears seven teeth; broad and elongated shape with two processes: laterally, there is a maxillary process, with wide basis and tapered end posteriorly curved and extending from teeth 5–7 (mp, Fig 14a), and medially the choanal process, broad and ventrally concave, situated after the last tooth to the end of the bone, not contacting parabasisphenoid (cp, Fig 14a); posterior edge single. *Ectopterygoids*: located on the mesolateral portion of the skull (Fig 11b); contacts maxilla on its anterior portion and pterygoid at posterior portion; elongated shape with anterior edge with expanded bifurcation and posterior edge unique; expanded portion corresponds to about one third of its total extension; in dorsal view, lateral branch of bifurcation has a small lateral process (Fig 13); first third of the bone, from its posterior end, contacts pterygoid, displaying less than half of the extension of pterygoid. *Pterygoids*: elongated, located on the posterior portion of palatomaxillary apparatus at the ventral face of the braincase (Fig 11b); contacts palatines on its anterior portion and ectopterygoid at mesolateral portion; bears 28 posteriorly curved teeth, being the anterior tooth larger than the posterior ones; in ventral view, anterior portion tapered, getting wider abruptly on the level of the 13^th^ tooth, where it contacts ectopterygoid (Fig 11b); gets broad again after the end of the teeth row, curving laterally (Fig 11b); small lateral process on the pterygoid-ectopterygoid joint; anterior edge simple; pterygoid extension corresponding to more than half of the whole skull extension; in dorsal view bears a lateral depression, from the articulation with ectopterygoid to edge of the bone.

SUSPENSORIUM AND MANDIBLE: *Supratemporals*: located on the dorsoposterior portion of the skull (Fig 11); overlaps much of the dorsal surface of the prootic and anterolateral portion of exoccipitals, contacting the most lateral part of supraoccipital; elongated shaped and dorsoventral compressed; posterior boundary beyond the posterior limit of the braincase. *Quadrates*: articulating with supratemporals anterodorsally and with the glenoid cavity of the compound bone posteroventrally (Fig 11a and 11c); bears a small mesomedial process in posterior view, the articulatory process of quadrate, which articulates with the *columella auris*; approximately triangular in lateral view, with dorsal portion straight and ventral portion tapered; about the same width in all its extension on posterior view; small anterodorsal process in contact with supratemporal. *Columella auris*: small, located on the lateroposterior portion of the braincase (Fig 11c); articulates with exoccipital through the foramen oval; formed by an anterior portion, round and expanded, which is inserted in the foramen oval, and an elongated and tapered region, extending towards quadrate. *Mandible*: composed of two sets of bones, the hemimandibles, those are arch-shaped. *Dentaries*: located in the anterior tip of the hemimandibles, posteriorly contacting angular and splenial in medial view and compound bone in lateral view; medially arched and elongated shaped; in lateral view bifurcated in its posterior edge, forming the dorsal and ventral processes of dentary; dorsal process longer than ventral; dorsal surface with 24 posteriorly curved teeth, being the anterior teeth longer than the posterior; dorsal process extending from tooth 14–24^th^; ventral process in the level of teeth 14–21^th^; in medial view, splenial overlaps ventral process, being visible only anterior portion of the bone and its dorsal process; meckel canal located between the ventral surface of the dorsal process and splenial; mental foramen on the level of 10–11^th^ teeth. *Splenials*: located in the posteroventral portion of dentary in lateral view; contacts angular posteriorly; triangular-shaped, with anterior edge tapered and posterior straight; bears the anterior mylohyoid foramen close to the joint with angular; about same extension of angular, but broader; small tapered process on the contact with dorsal portion of angular-splenial joint; posterior limit on the level of dentary-compound bone suture. *Angulars*: located in the posterior portion of splenial in medial view; contacts splenial anteriorly, dentary anteriorly (dorsally and ventrally), and compound bone along all its extension; bears the posterior mylohioyd foramen on its mesoanterior portion; triangular shaped, with anterior edge straight and posterior tapered; anterior limit on the level of dentary-compound bone suture; posterior boundary surpasses the posterior limit of dorsal process of dentary; small tapered dorsoanterior process, on the suture with splenial; angular-splenial suture visible in ventral view. *Compound bones*: represent the fusion between prearticular, articular, and surangular bones; largest bone of the mandible, located on its posterior portion, with elongated shape; contacts dentary and angular anteriorly, and articulates with quadrate posteriorly, through the glenoid cavity, a saddle-shaped cavity; in lateral view, anterior region projects between dorsal and ventral processes of dentary; in medial view, anterior edge projects dorsally to angular and ventrally to dorsal process of dentary; anteriorly to glenoid cavity, two crests are present: the prearticular and the surangular crests; in lateral view, prearticular crest slightly higher than surangular; between those crests there is a cavity that ends in its anterior portion with a foramen, the posterior orifice of the inferior dentary canal, which possesses a way out to the lateral face of the bone thought a foramen situated slightly posterior to the end of dorsal process of dentary (anterior surangular foramen); retroarticular process present.

**Fig 15:**
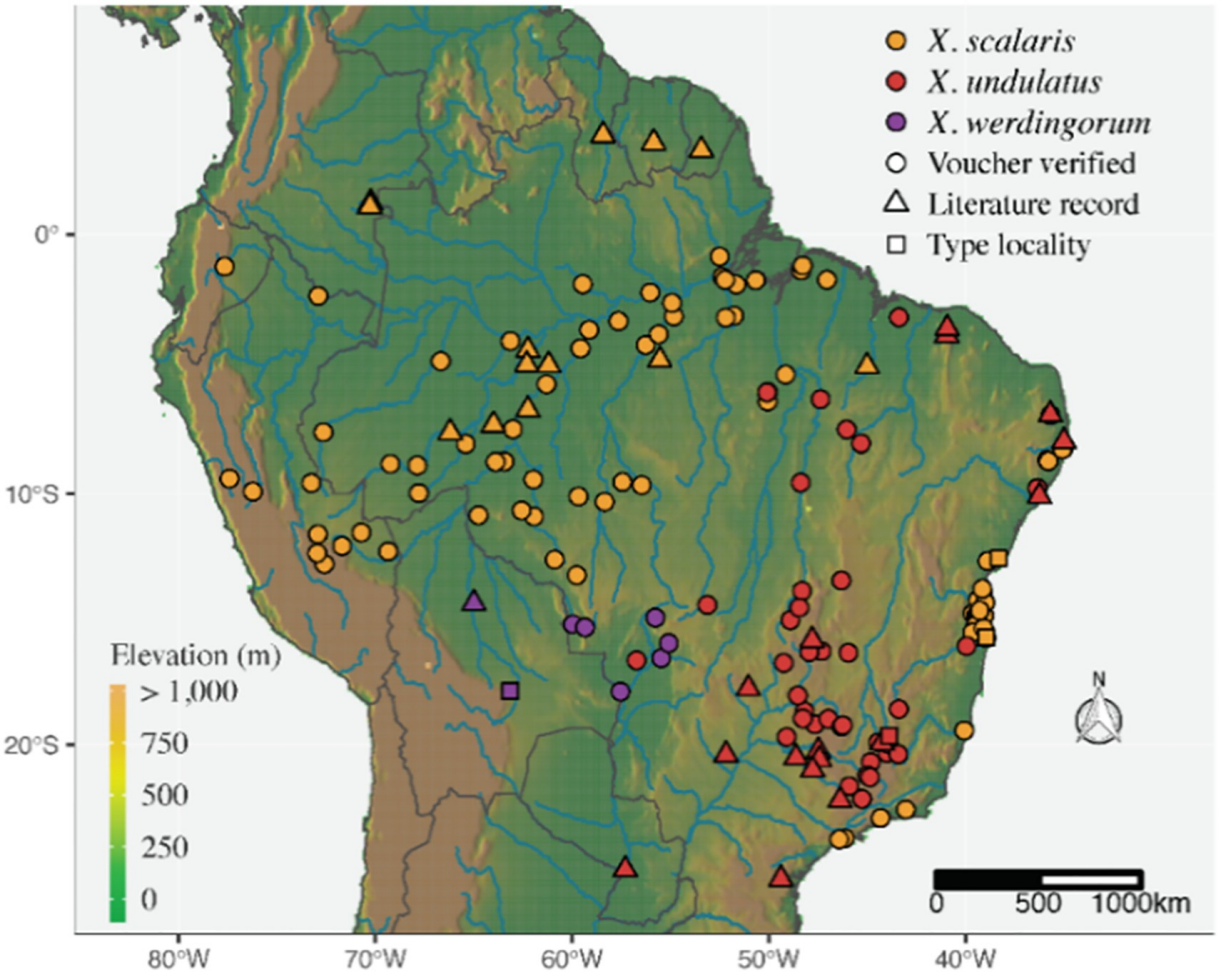
Known distribution of the genus *Xenopholis*. *Xenopholis undulatus* is mostly distributed in riparian forests of the Cerrado and Caatinga highlands, whereas *X*. *scalaris* is mostly distributed in lowland tropical forests. *Xenopholis werdingorum* is distributed mostly within the Pantanal wetlands and in the Chiquitanos forests. Digital elevation model source: Global Multi-resolution Terrain Elevation Data 2010 (GMTED2010). Earth Resources Observation and Science (EROS) Center (open source). DOI: 10.5066/F7J38R2N.

*Distribution (*Fig 15*)*. Based on available records obtained by examination of preserved samples, literature data, and environmental niche models, *Xenopholis scalaris* is restricted to lowland ombrophilous forest from east Andes. This species is distributed in the Amazonia domain in Bolivia, Brazil, Colombia, Ecuador, French Guiana, Guyana, Peru, and Suriname, with disjunct populations along the Brazilian Atlantic Forest from Pernambuco to Rio de Janeiro States (Fig 12). Mumaw [70] included this species in the snake fauna of Venezuela based on the AMNH-R 4443 specimen. However, such a record is doubtful since the location for this specimen is Brazil-Venezuela, with no additional information. Even though the occurrence of this species in the Amazonian portion of Venezuela is very likely, as far we know, there is no voucher of *X*. *scalaris* with precise provenance came from Venezuela. Therefore, considering only the accurate data available, we exclude *X*. *scalaris* from the confirmed Venezuelan snake fauna.

#### *Xenopholis undulatus* (Jensen, 1900)

*Oxyrhopus undulatus* Jensen, 1900. Videnskabelige meddelelser fraden Naturhistoriske forening i Kjobenhavn, 1900:106. (two syntypes collected by Prof. Reinhardt and Prof. E. Warming from Lagoa Santa 19°38'S 43°53'W, state of Minas Gerais, Brazil).

*Paroxyrhopus reticulatus* Schenckel, 1901. Verh. Naturforsh. Ges. Basel 13:169. (holotype female from Bernal-Cué 25°15’S 57°17’W, Paraguay).

*Oxyrhopus latifrontalis* Werner, 1913. Mitt. Naturhist. Mus. Hamburg 30:30. (holotype male collected at the eastern portion of the state of Minas Gerais, Brazil).

*Paroxyrhopus atropurpureus* Amaral, 1923. Proc. New England Zool. Club. Vol.8:90. (holotype adult male IBSP 3003 from Nova Baden 19°58'S 44°6'W, state of Minas Gerais, Brazil; paratype adult male MZUSP 1499 from a locality near Mariana, state of Minas Gerais, Brazil).

*Paroxyrhopus latifrontalis*—Amaral, 1930. Mem. Inst. Butantan 4:208. (holotype, HMZ 4811, from the west of Minas Gerais State).

*Paroxyrhopus undulatus* Bailey in Peters & Orejas Miranda, 1970. Cat. Neotrop. Squamata Snake:238.

*Paroxyrhopus reticulatus* Bailey in Peters & Orejas Miranda, 1970. Cat. Neotrop. Squamata Snake:238.

*Comparative diagnosis*. *Xenopholis undulatus* can be distinguished from all congeners by the following characters: (1) dorsum of head black in life and after preservation (vs. dorsum of head red to reddish-brown in life and brown or pale brown in preservative in *X*. *scalaris*); (2) dorsal ground color covered with a conspicuous black, broad and irregular vertebral stripe (vs. dorsal ground color of body red, reddish brown to orange in life, and light or pale brown after preservation, with black alternated paravertebral blotches, sometimes connected forming conspicuous cross-bands in *X*. *scalaris*. Dorsum black with three paraventral scale rows orange in life and pale brown after preservation in *X*. *werdingorum*); (3) dorsal scales rows 19/19/17 (vs. 17/17/17 in *X*. *scalaris*); (4) ventral scales 160–190 in males, 168–196 in females (vs. 126–169 in males of *X*. *scalaris* and 181–195 of *X*. *werdingorum*, 128–175 in females of *X*. *scalaris* and 180–196 of *X*. *werdingorum*); (5) subcaudal scales 36–55 in males, 33–60 in females (vs. subcaudal scales 28–45 in males of *X*. *scalaris* and 46–54 of *X*. *werdingorum*, 27–42 females of *X*. *scalaris* and 38–48 of *X*. *werdingorum*); (6) two postocular (postocular single in *X*. *scalaris*); (7) hemipenis unilobed usually with single sulcus spermaticus (vs. hemipenis unilobed with bifurcated sulcus spermaticus in *X*. *scalaris* and bilobed in *X*. *werdingorum*); (8) hemipenis slightly capitulated (vs. hemipenis strongly capitulated on the sulcate side in *X*. *scalaris* and not capitulated in *X*. *werdingorum*); (9) capitulum ornamented with papillae on the distal portion of capitulum (vs. capitulum ornamented with spinulate calyces in *X*. *scalaris* and entirely papillate in *X*. *werdingorum*); (10) hemipenial body ornamented with hooked spines and dispersed papillae (vs. hemipenial body ornamented with hooked spines and longitudinal plicae in *X*. *scalaris* and hemipenial body ornamented with lateral spines and dispersed papillae in *X*. *werdingorum*); (11) pupil brown (vs. pupil red in *X*. *scalaris*); (12) neural spine of vertebrae with a narrow septum perpendicular to longitudinal axis of body (vs. absent in *X*. *scalaris*); (13) vomerian process of premaxillae overlapping vomers (vs. contacting anteromedial portion of vomers in *X*. *scalaris*); (14) nasal process absent (vs. present in *X*. *scalaris* and *X*. *werdingorum*); (15) pair of nasals smaller than frontals (vs. about the same length of frontals in *X*. *scalaris*); (16) dorsal crests of parietal not contacting each other (vs. contacting each other in *X*. *werdingorum*); (17) contact between frontals and postorbitals (vs. no contact in *X*. *scalaris*); (18) contact between supratemporals and supraoccipital absent (vs. present in *X*. *scalaris* and *X*. *werdingorum*); (19) ten palatine teeth (vs. seven in *X*. *scalaris*); (20) 14 teeth in the pterygoids (vs. 28 in *X*. *scalaris* and 23 in *X*. *werdingorum*).

**Fig 16:**
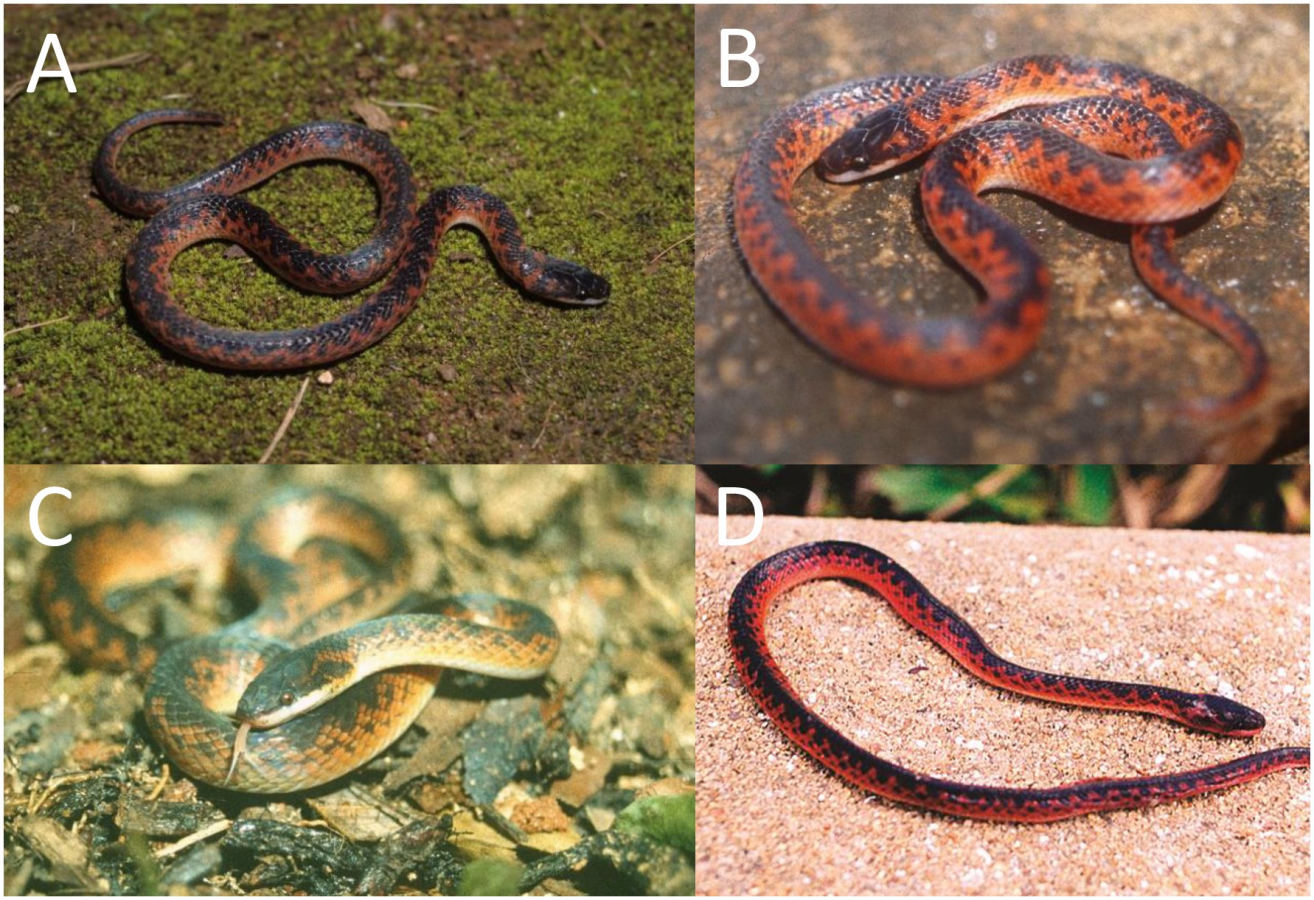
Color variability of *Xenopholis undulatus* in life. A—Palmas, state of Tocantins, Brazil; B—Lajeado, state of Tocantins, Brazil; C—Lindóia, state of São Paulo, Brazil; D—Salto da Divisa, state of Minas Gerais, Brazil. Photos by O. Marques (A); M. R. Duarte (B, C); M. A. Freitas (D).

*Color pattern in life (*Fig 16*)*. Dorsal part of the head almost entirely black, except for irregular red spot(s) or blotch(es) covering the parietal and/or the occipital region; lateral surface of the head black on the dorsal edges of the supralabials; supralabials usually uniformly creamish white, sometimes with invasion of black pigment; dorsal ground color of body red to reddish-orange, except for the first one or two scale rows, which are creamish white or reddish cream; dorsum with a conspicuous and winding vertebral stripe, extending from the cephalic-cap to the tip of the tail; vertebral stripe with lateral projections in zig-zag or symmetrical expansion to the paravertebral region; lateral expansion reaching seventh or eighth scales rows direct to ventral surface of body; between third to sixth or seventh dorsal scale rows there are black spots or blotches (half-scale to two scales long) on the interspaces among the lateral expansion of vertebral stripe; sometimes those black marks connect to a lateral expansion along the body extension, giving impression of an irregular dorsal pattern; ventral surface of body uniformly creamish white to reddish cream. Iris brown.

*Color variability observed in preserved specimens (*S2 Fig*)*. The color pattern after preservation is very similar to coloration in life, only changing to fading red, orange, and reddish-orange pigments. The orange and reddish-orange or red pigments become pale brown and brown, respectively.

*Quantitative variability for secondarily dimorphic characters*. Number of ventral scales 160–190 (mean = 178.86, SD = 7.3, N = 22) in males, 168–196 (mean = 181.02, SD = 6.35, N = 49) in females, number of subcaudal scales 36–55 (mean = 44.59, SD = 5.52, N = 22) in males, 33–60 (mean = 41.46, SD = 5.22, N = 49) in females, and number of preoculars 1 (N = 22) in males, 1–2 (mean = 1.04, SD = 0.20, N = 48) in females. Variables that did not present sexual dimorphism are presented on Table 4.

**Table 4:** Selected variables synthesizing the meristic and morphometric variation of *Xenopholis undulatus*.

	Min	Max		SD	-95%	95%	N
SVL (mm)	134	390	267.94	63.69	252.86	283.01	71
CL (mm)	21	69	47.76	12.47	44.80	50.71	71
TL (mm)	156	452	315	75.19	297.97	333.57	71
Distance nostril (mm)	1.16	2.95	2.19	0.42	2.09	2.29	67
Eye circumference (mm)	1.00	1.80	1.37	0.16	1.33	1.41	68
Distance nostril-eye (mm)	1.47	3.60	2.34	0.43	2.23	2.44	67
Distance rostral-eye (mm)	2.23	4.61	3.52	0.54	3.38	3.65	67
Distance eye (mm)	2.54	4.90	3.50	0.50	3.38	3.63	68
Head length (mm)	8.18	14.74	11.64	1.75	11.22	12.07	67
Head width (mm)	4.10	7.80	5.78	0.74	5.54	6.01	68
Head height (mm)	2.63	5.33	3.87	0.65	3,71	4.03	67
Dorsal I	19	19	19	0	-	-	71
Dorsal II	19	19	19	0	-	-	71
Dorsal III	17	17	17	0	-	-	71
First temporal	1	1	1	0	-	-	70
Second temporal	2	2	2	-	-	-	70
Third temporal	2	3	2.84	0.36	2.75	2.93	70
Supralabial	7	8	7.98	0.11	7.95	8.01	70
Larger supralabial	6	7	6.67	0.47	6.55	6.78	70
Geniais	4	4	4	0	-	-	71
Infralabial	8	9	8.95	0.20	8.90	9.00	70
1° supralabial-eye	3	4	3.97	0.16	3.93	4.01	70
2° supralabial-eye	4	5	4.95	0.20	4.90	5.00	70
Pos-ocular	1	2	1.02	0.16	0.98	1.06	70
IL cont. 1° ment.	1	4	-	-	-	-	70
IL cont. 2° ment.	4	5	-	-	-	-	70
Prefrontal	1	2	1.98	0.11	1.95	2.01	70
Maxillary teeth	11	12	11.84	0.36	11.75	11.93	64
Number of spots	35	79	69.32	5.90	67.92	70.72	71

Abbreviations are as follow: CL = caudal Length; SVL = snout-vent length; TL = total length; IL cont. 1°/2° ment. = infralabial contact with the first/second mentonian; Max. = maximum; Min. = minimum; N = sample size; SD = standard deviation; -95% = lower limit of the confidence interval; + 95% = upper limit of the confidence interval.

**Fig 17:**
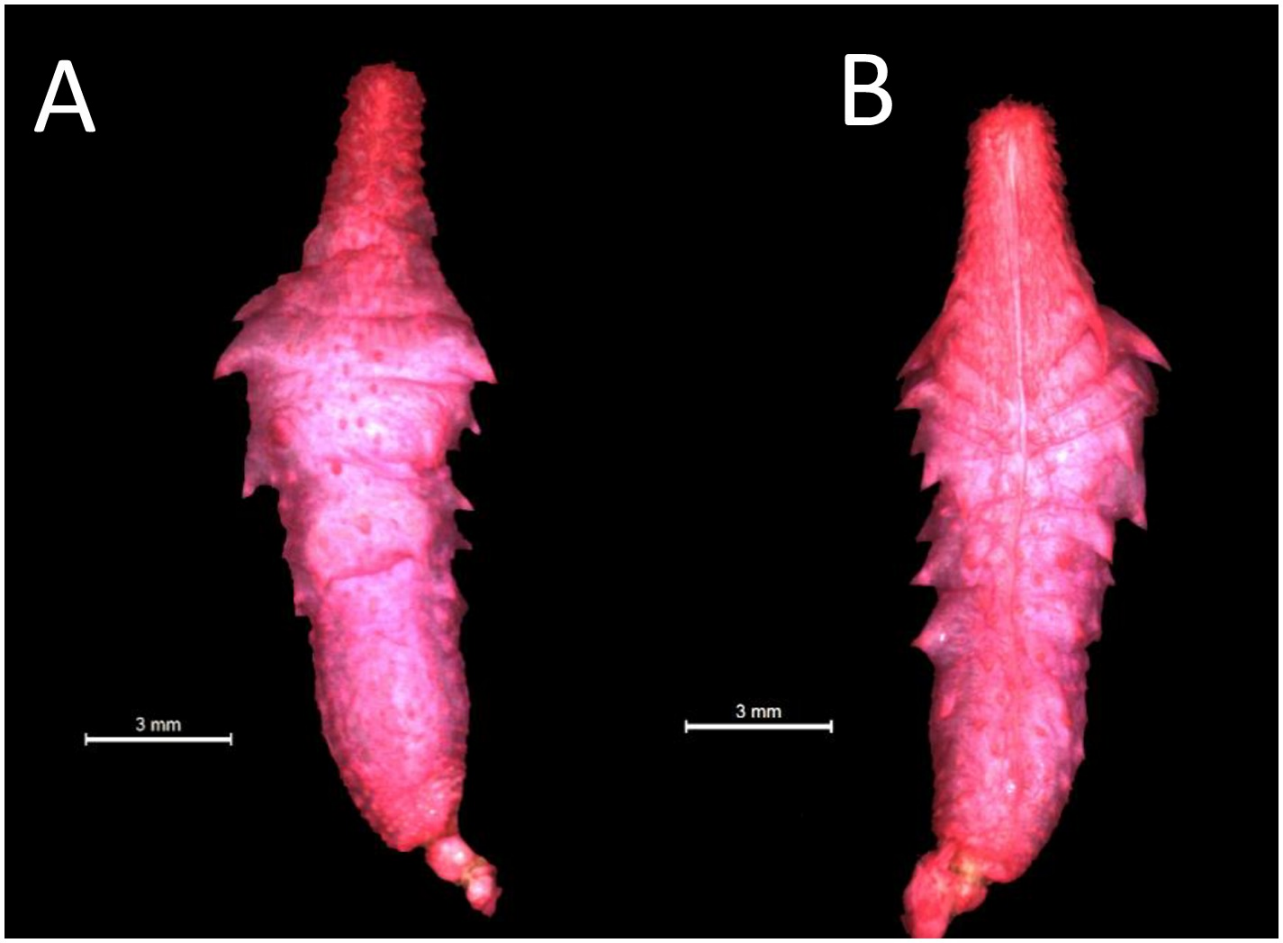
Asulcate (A) and sulcate (B) sides of the hemipenis of *Xenopholis undulatus* from state of Minas Gerais, Brazil (FUNED 2180).

*Hemipenial morphology (*Fig 17*)*. Fully everted and maximally expanded hemipenes rendered an unilobed, unicalyculate, and weakly semicapitate organ; capitulum slender than hemipenial body; capitular crotch barely distinct on the asulcate side and nearly indistinct at sulcate face of hemipenis; capitulum attenuated and shorter than hemipenial body; capitulum uniformly covered by papillate calyces; on the sulcate face of the organ there are two rows of lateral hooked spines inside the capitulum area; basal region of capitulum on the sulcate and lateral faces with hooked spines arranged approximately in traversal rows; hemipenial body on the sulcate side with about six to seven lateral hooked spines, three distal rows following shallow transversal grooves; hemipenial body elliptical and scattered with large hooked spines and disperse papillae; asulcate side of hemipenis with four rows of hooked spines, transversally arranged into three shallow grooves; lateral hooked spines decreasing in size on both sides of organ, from the capitulum toward the basal region of hemipenial body; hemipenial body with papillae among hooked spines on both sides of the organ; larger spines located laterally below capitulum; sulcus spermaticus usually single and running to distal region of capitulum, but not reaching it edge; sometimes sulcus spermaticus bifurcates on distal region of capitulum (cf. Zaher, 1999) [40]; sulcus spermaticus margins expanded inside capitulum, and not bordered by some papillae along hemipenial body; proximal region of hemipenial body with high concentration of papillae; basal naked pocket absent or indistinct.

**Fig 18:**
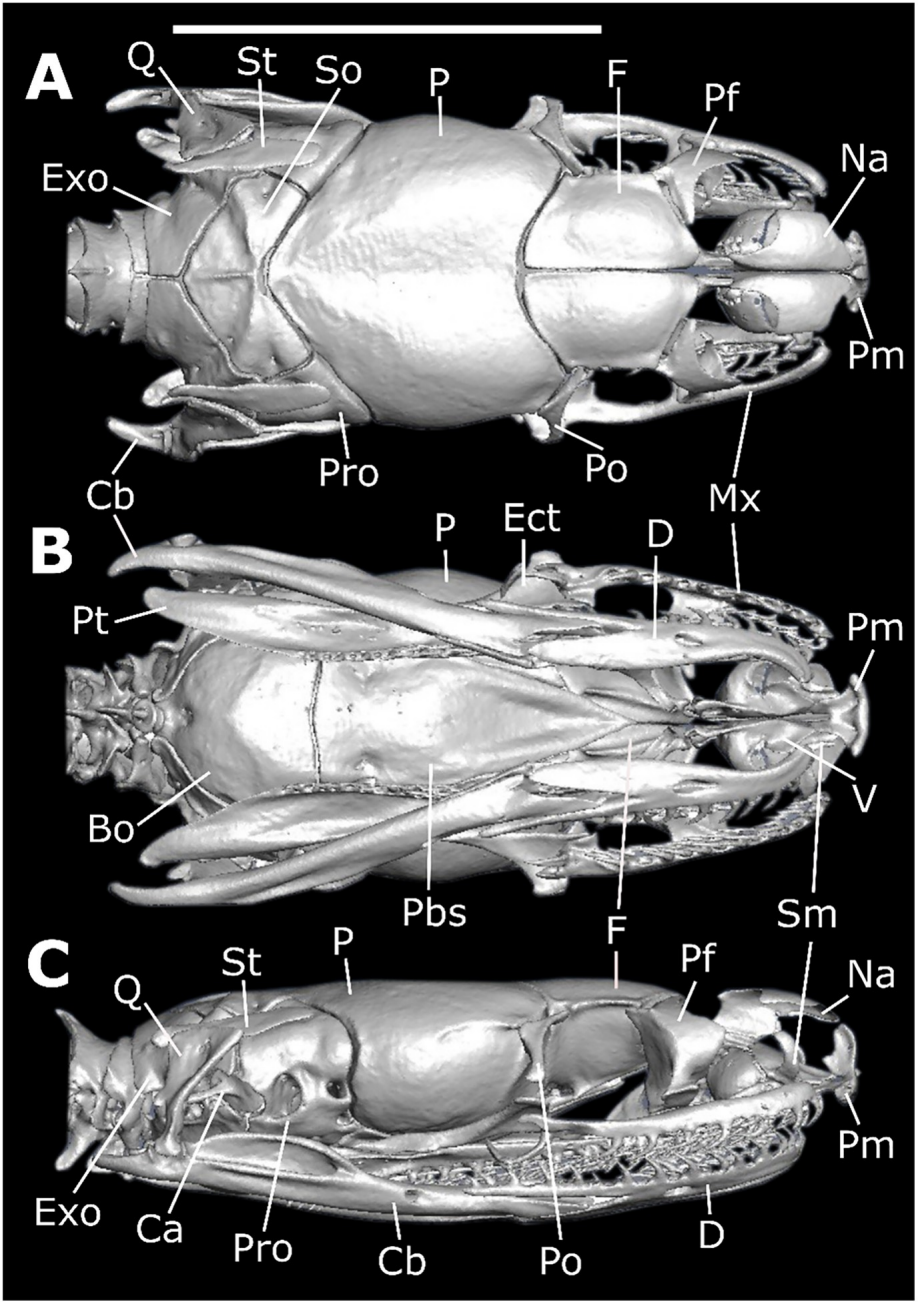
Dorsal (A), ventral (B), and lateral (C) views of the skull of *Xenopholis undulatus* (UMMZ 108820) from Lajeado, state of Tocantins. Abbreviations are as follow: Cb = compound bone; So = supraoccipital; Pro = prootic; P = parietal; F = frontal; Mx = maxilla; Na = nasal; Pm = premaxilla; Pf = prefrontal; Po = postorbital; Ect = ectopterygoid; St = supratemporal; Q = quadrate; Pt = pterygoid; Exo = exoccipital; V = vomer; Sm = septomaxilla; Pbs = parabasisphenoid; Bo = basioccipital; Ca = *columella auris*; and D = dentary.

*Skull morphology*. The cranium of *Xenopholis undulatus* (Fig 18) is very similar to the cranium of *X*. *scalaris*; differences are summarized on Table 6.

*Distribution (*Fig 15*)*. *Xenopholis undulatus* is restricted to highland portions of the Caatinga, ecotonal zones between Caatinga and Atlantic Forest, and to riparian forests across the Cerrado. This species has a widespread distribution along the Brazilian Shield from the Maranhão to the Paraná States, reaching the Paraguayan Chaco on the west side of Paraná River.

#### *Xenopholis werdingorum* Jansen, Álvarez & Kohler, 2009

*Xenopholis* sp.—Marques, Eterovic, Strüssmann & Sazima, 2005:73 [71] (Cabaceiras Farm, municipality of Poconé, 16°15’24”S 56°37’22”W, state of Mato Grosso, Brazil).

*Comparative diagnosis*. *Xenopholis werdingorum* can be distinguished from all congeners by the following characters: (1) dorsum of the head black in life and after preservation (vs. reddish-brown in *X*. *scalaris*); (2) dorsum of the body black with three paraventral scale rows, orange in life and pale brown after preservation (vs. red dorsal ground color, reddish brown to orange in life and light or pale brown after preservation, with black alternate paravertebral blotches, sometimes connected forming conspicuous cross-bands in *X*. *scalaris*, and dorsum covered with a conspicuous black, broad and irregular vertebral strip in *X*. *undulatus*); (3) dorsal scales rows 19/19/17 (vs. 17/17/17 in *X*. *scalaris*); (4) ventral scales 181–195 in males, and 180–196 in females (vs. 126–169 in males and 128–175 in females of *X*. *scalaris*, and 160–190 in males and 168–196 in females of *X*. *undulatus*); (5) subcaudal scales 46–54 in males, and 38–48 females (vs. 28–45 in males and 27–42 in females of *X*. *scalaris*, and 36–55 in males and 33–60 in females of *X*. *undulatus*); (6) two postocular scales (vs. postocular single in *X*. *scalaris*); (7) hemipenis bilobed (vs. hemipenis unilobed with bifurcated sulcus spermaticus in *X*. *scalaris* and unilobed usually with single sulcus spermaticus in *X*. *undulatus*); (8) hemipenis not capitulated (vs. hemipenis strongly capitulated on the sulcate side in *X*. *scalaris* and little capitulated in *X*. *undulatus*); (9) capitulum entirely papillate (vs. capitulum ornamented with spinulate calyces in *X*. *scalaris*, and papillate on distal portion of capitulum in *X*. *undulatus*); (10) hemipenial body ornamented with lateral spines and dispersed papillae (vs. hemipenial body ornamented with hooked spines and longitudinal plicae in *X*. *scalaris*, and hemipenial body ornamented with hooked spines in *X*. *undulatus*); (11) iris brown (vs. pupil red in *X*. *scalaris*); (12) neural spine of vertebrae with a narrow septum perpendicular to the longitudinal axis of body (vs. septum absent in *X*. *scalaris*); (13) vomerian process of premaxillae overlapping vomers (vs. contacting anteromedial portion of vomers in *X*. *scalaris*); (14) nasal process present (vs. absent in *X*. *undulatus*); (15) pair of nasals smaller than frontals (vs. about the same length of frontals in *X*. *scalaris*); (16) dorsal crests of parietal contacting each other (vs. not contacting each other in *X*. *scalaris* and *X*. *undulatus*); (17) contact between frontals and postorbitals present (vs. no contact in *X*. *scalaris*); (18) contact between supratemporals and supraoccipital present (vs. absent in *X*. *undulatus*); (19) ten palatine teeth (vs. seven in *X*. *scalaris*); (20) 23 teeth in the pterygoids (vs. 28 in *X*. *scalaris* and 14 *X*. *undulatus*).

**Fig 19:**
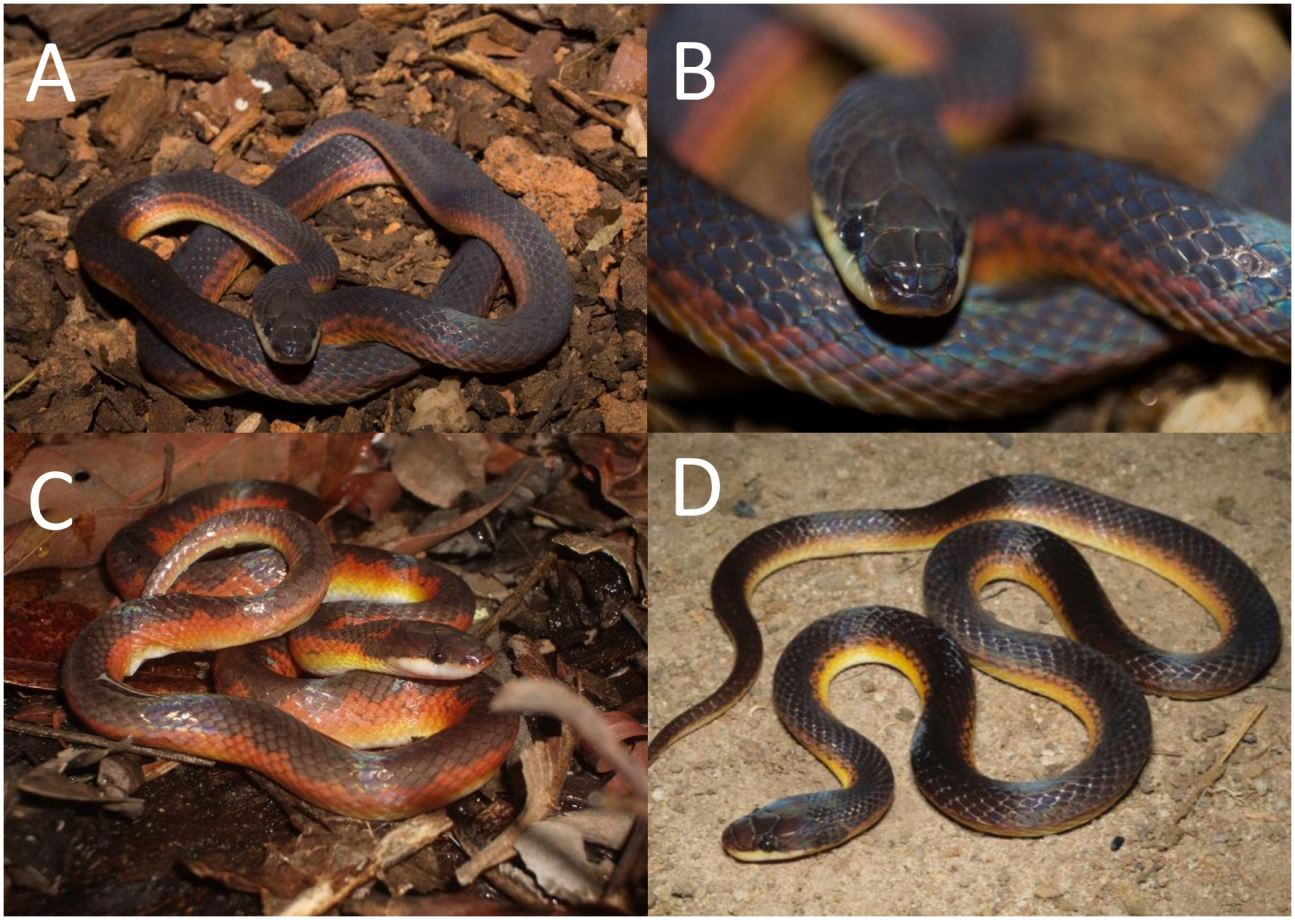
Color variability of the *Xenopholis werdingorum* in life. A–B Serra de São Vicente, state of Mato Grosso, Brazil; C—Pirizal, Nossa Senhora do Livramento, state of Mato Grosso, Brazil; D—Poconé, state of Mato Grosso, Brazil. Photos by A. Andrade-Jr. (A–B); C. Strussman (C) and O. Marques (D).

*Color pattern in life (*Fig 19*)*. Dorsum and background of head uniformly black to the dorsal margins of the supralabials; supralabials creamish white; ventral surface of the body creamish white to creamish yellow; dorsal ground color of the body mostly black, except for the first five to six scale rows red, orange or yellow colored; first scale row usually creamish white, followed for two or three yellow scale rows or five red to orange-red scale rows; more rarely, the black area may be restricted to seven to eight vertebral or paravertebral scale rows, resembling the winding vertebral stripe of the *X*. *undulatus*. Iris brown.

*Color variability observed in preserved specimens (*S3 Fig*)*. The color pattern after preservation is very similar to coloration in life, only changing to fading red, reddish-orange, and yellow pigments. The reddish-orange and red pigments become pale brown and brown, respectively; while yellow and creamish yellow pigments become cream.

*Quantitative variability for secondarily dimorphic characters*. Caudal length 58–82 in males (mean = 67.80, SD = 8.72, N = 5), 25–62 in females (mean = 48.57, SD = 13.86, N = 7). Variables that did not present sexual dimorphism are presented on Table 5.

**Table 5:** Selected variables synthesizing the meristic and morphometric variation of *Xenopholis werdingorum*.

	Min	Max		SD	-95%	95%	N
SVL (mm)	160	370	298.50	62.80	258.58	338.42	12
CL (mm)	25	82	56.58	15.18	46.93	66.23	12
TL (mm)	185	452	355.08	76.58	306.42	403.74	12
Distance nostril (mm)	1.34	3.23	2.41	0.51	2.09	2.74	12
Eye circumference (mm)	1.12	1.76	1.48	0.18	1.36	1.59	12
Dist. nostril-eye (mm)	1.93	3.91	2.58	0.50	2.26	2.90	12
Dist. rostral-eye (mm)	2.72	4.64	3.73	0.49	3.41	4.04	12
Dist. eye (mm)	2.86	4.73	3.70	0.48	3.40	4.01	12
Head length (mm)	8.46	15.00	12.44	1.64	11.39	13.48	12
Head width (mm)	4.42	7.72	6.36	0.81	5.84	6.87	12
Head height (mm)	2.86	4.83	3.95	0.58	3.58	4.32	12
Dorsal I	19	19	19	0	-	-	12
Dorsal II	19	19	19	0	-	-	12
Dorsal III	17	17	17	0	-	-	12
First temporal	1	1	1	0	-	-	11
Second temporal	2	2	2	-	-	-	11
Third temporal	1	1	1	0	-	-	11
Supralabial	8	8	8	-	-	-	11
Larger supralabial	6	7	6.81	0.40	6.54	7.08	11
Geniais	4	4	4	-	-	-	11
Infralabial	8	9	8.90	0.30	8.70	9.11	11
1° supralabial-eye	4	4	4	-	-	-	11
2° supralabial-eye	5	5	5	-	-	-	11
Pos-ocular	2	2	2	-	-	-	10
Prefrontal	2	2	2	-	-	-	12
Maxillary teeth	11	12	11.83	0.38	11.58	12.08	12
Number of spots	-	-	-	-	-	-	12

Abbreviations are as follow: CL = caudal length; SVL = snout-vent length; TL = total length; IL cont. 1°/2° ment. = Infralabial contact with the first/second mentonian Max. = maximum; Min. = minimum; N = sample size; SD = standard deviation; -95% = lower limit of the confidence interval; + 95% = upper limit of the confidence interval.

**Fig 20:**
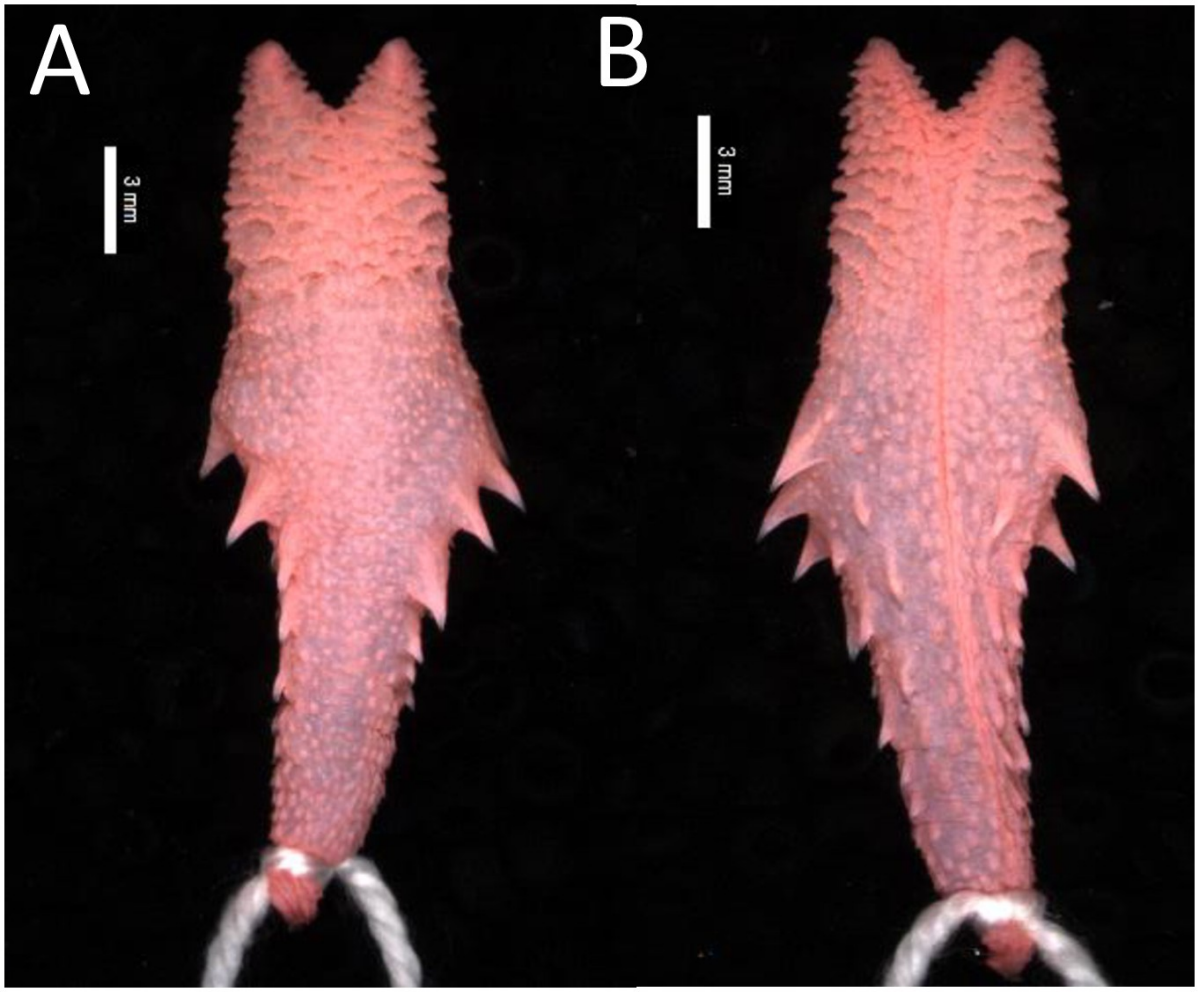
Asulcate (A) and sulcate (B) side of the hemipenis of *Xenopholis werdingorum* from Corumbá state of Mato Grosso do Sul, Brazil (UFMT-R 1193).

*Hemipenial morphology (*Fig 20*)*. Fully everted and maximally expanded hemipenes rendered a moderately bilobed, bicalyculate and non-capitate organ; capitulum with similar width than distal portion of hemipenial body; capitular crotch indistinct on both faces of organ; labels attenuate and shorter than the remaining capitular region; capitulum with approximately half-size of the hemipenial body; capitulum uniformly covered by papillate calyces; calyces transversally arranged on the sulcate and lateral faces of hemipenis, and almost irregular on the asulcate side of organ; basal region of capitulum without hooked spines delimiting capitulation region on both sides of hemipenis; hemipenial body elliptical with a narrowing portion toward proximal region of hemipenis; narrowing region delimited by large hooked spines, which are concentrated on lateral region of hemipenial body; hemipenial body on the asulcate side of hemipenis entirely covered with high concentration of papillae; hemipenial body on the sulcate face of organ ornamented with papillae and two longitudinal rows of hooked spines; each longitudinal row placed on one side of sulcus spermaticus, displaying about three or four mid-sized hooked spines; sulcus bifurcates on distal third of organ within capitulum, with each branch centrolinearlly oriented extending to edges of lobes; sulcus spermaticus margins narrow along all its extension, and bordered by papillae; basal naked pocket absent or indistinct; most basal region of hemipenis entirely covered by high concentration of papillae.

**Fig 21:**
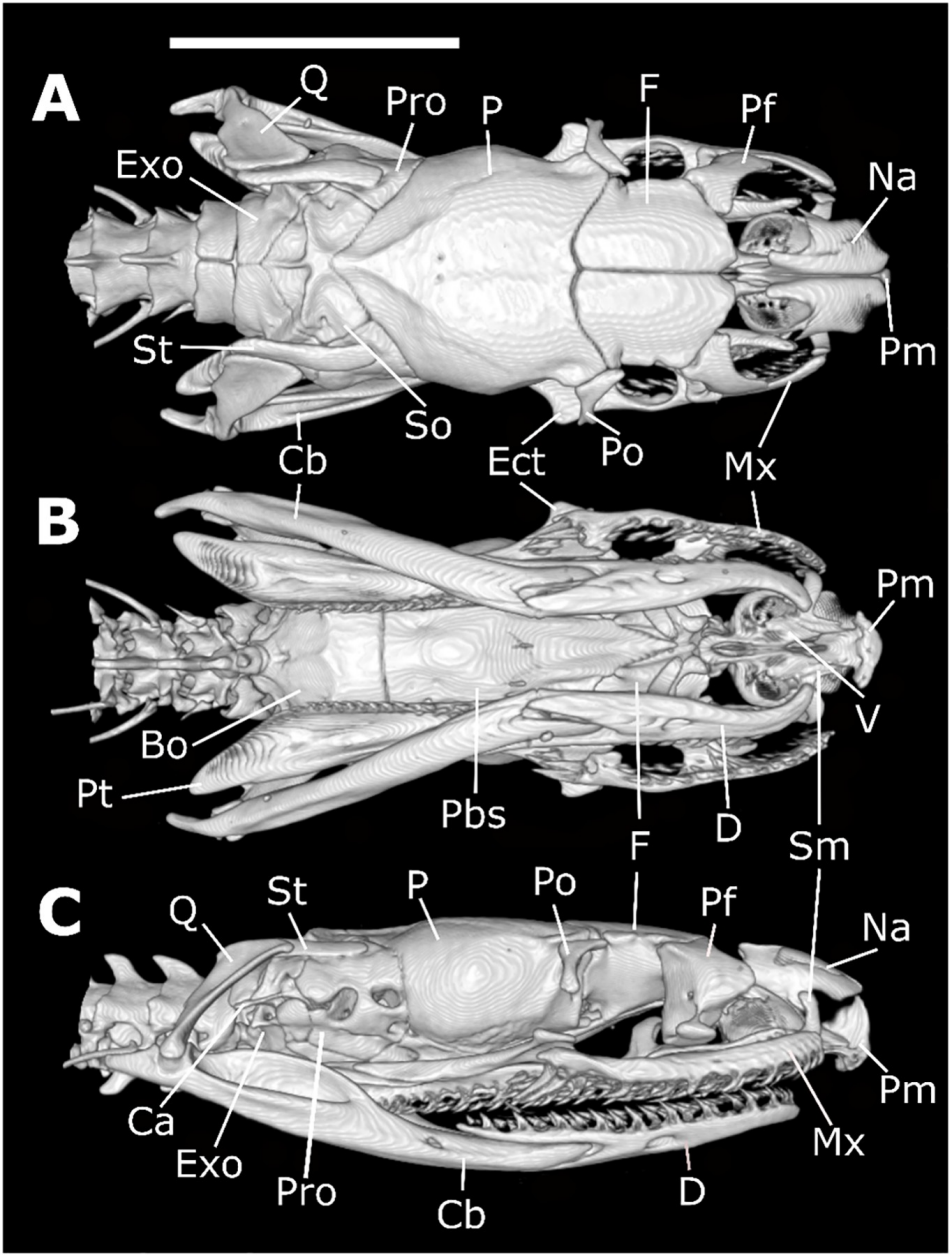
Dorsal (A), ventral (B), and lateral (C) views of the skull of *Xenopholis werdingorum* (UFMT-R 12051) from Santo Antônio do Leverger, state of Mato Grosso. Abbreviations are as follow: Cb = compound bone; So = supraoccipital; Pro = prootic; P = parietal; F = frontal; Mx = maxilla; Na = nasal; Pm = premaxilla; Pf = prefrontal; Po = postorbital; Ect = ectopterygoid; St = supratemporal; Q = quadrate; Pt = pterygoid; Exo = exoccipital; V = vomer; Sm = septomaxilla; Pal = palatine; Pbs = parabasisphenoid; Bo = basioccipital; Ca = *columella auris*; and D = dentary.

*Skull morphology*. The cranium of *Xenopholis werdingorum* (Fig 21) is very similar to the cranium of *X*. *scalaris*; differences are summarized on Table 6.

**Table 6:** Comparison of the cranial morphology in the three species of the genus *Xenopholis*.

Characters/Species	*X*. *scalaris*	*X*. *undulatus*	*X*. *werdingorum*
**Premaxilla**			
Ascendant process	With a pair of lateral projection on its base; base wider than dorsal edge; dorsal edge tapered	No pair of lateral projection on its base; same width in all extension; blunt edge	With a pair of lateral projection on its base; broad; similar width in all its extension; blunt edge
Vomerian processes	Divergent; contacting anteromedial portion of vomers	Lateral lamina convergent and medial parallel; overlapping vomer	Lateral lamina convergent and medial parallel; overlapping vomer
Nasal process	Present	Absent	Present
**Septomaxillae**			
Conchal process	Tapered edge posteriorly turned	Tapered, but blunt edge, posteriorly turned	Tapered edge posteriorly turned
Contact of conchal process and nasal	Present	Present	Absent
**Vomers**			
Anterior process	Lateral to vomerian process of premaxilla, contacting it	Overlapped by vomerain process of premaxilla	Overlapped by vomerain process of premaxilla
Posterior process	Small foramen in its ventral portion	Medium size foramen in its ventral portion	Large size foramen in its ventral portion—occupying half of its high
Mesolateral projection	Not overlapped by palatines	Not overlapped by palatines	Slightly overlapped by palatines
**Nasals**			
Size in dorsal view	Large—about the same extension of frontals	Small—smaller than frontals	Small—little smaller than frontals
**Frontals**			
Size in dorsal view	About the same extension of nasals and half extension of parietal	Larger than nasals; about 2/3 of parietal extension	Larger than nasals
Contact with postorbital	Absent	Present—posterolateral portion	Present—posterolateral portion
Optical foramen	Totally inserted in the frontals	Inserted equally in the frontals and parietal	Inserted equally in the frontals and parietal
**Parietal**			
Shape of posterior edge	Rounded	Rounded	Tapered
Suture with frontal	Convex	Almost straight and oblique to the lateromedial axis (concave aspect)	Convex
Dorsal crests	Not contacting each other	Not contacting each other	Contacting each other on its posterior edge
**Supraoccipital**			
Contact with supratemporals	Present	Absent	Present
Foramina in dorsal view	Two pairs of small foramina on the mesolateral portion	No visible foramina	Two pairs of small foramina on the mesolateral portion
**Exoccipitals**			
Foramen oval	Not located in the suture with prootic	Located on its anteromesial portion, in the suture with prootic in lateral view.	Located on its anteromesial portion, in the suture with prootic in lateral view.
Other foramina	Present ventral to the foramen oval	No visible foramina ventral to the foramen oval	Present ventral to the foramen oval
Process in the suture with basioccipital	Absent	Present	Present
**Basioccipital**			
Suture with parabasisphenoid	Straight	Slightly concave	Straight
Sutures with exoccipitals	Mesolateral process absent	Mesolateral process slightly developed	Mesolateral process developed on its anterior portion
Dentigerous process	Two forming a slightly developed crest on its mesial portion	Three, forming a slightly developed crest on its mesial portion that continues until the mesolateral processes	Three, forming a slightly developed crest on its mesial portion that continues until the mesolateral processes
**Prootics**			
Foramen oval	Not situated in the suture with exoccipital	Situated in the suture with exoccipital	Situated in the suture with exoccipital (in one side of the skull, the foramen connected to foramen for the mandibular branch of trigeminal)
**Prefrontals**			
Lateral view	Anterior portion with a convex projection and posterior portion concave	Anterior portion with a convex projection in the mesial region and concave lamina dorsal to it	Tapered portion right above its ventral edge
**Postorbitals**			
Contact with frontals	Absent	Present—most lateroposterior portion of it	Present—most lateroposterior portion of it
Shape	Subtriangular—dorsal edge straight and ventral tapered	Nearly “C”–anterior border concave and posterior convex	Nearly “C”–anterior border concave and posterior convex
Small posterior process on the most ventral point	Absent	Present	Present
**Maxillae**			
Contact with palatine	Absent	Absent	Present—palatine process overlaps the maxillary process of palatine
Number of prediastemal teeth	15	16	13
Size of grooved teeth	About the same size of prediastemal teeth	Larger than prediastemal teeth	Larger than prediastemal teeth
Location of palatine process	9–12 tooth	8–12 tooth	9–11 tooth
**Palatines**			
Number of teeth	7	10	10
Location of maxillary process	5–7 tooth	7–10 tooth	5–9 tooth
Location of choanal process	After the last tooth to the end of the bone	After the last tooth to the end of the bone	From 9 tooth to the posterior portion of the bone
**Pterygoids**			
Number of teeth	28	14	23
Location of wider portion	From tooth 13^th^	From tooth 13^th^	From tooth 12th
Lateral process in the pterygoid-ectopterygoid joint	Present—small	Absent	Present—small
Crest in dorsal view	Absent	Present from the articulation with ectopterygoid to the edge of the bone	Present from the articulation with ectopterygoid to the edge of the bone
**Ectopterygoid**			
Extension of expanded portion	About 1/3 of the bone	About 1/3 of the bone	About ¼ of the bone
Contact with pterygoid	First third of the bone	First third of the bone	First fourth of the bone
Size	Less than half of the pterygoid	Less than half of the pterygoid	About half of the pterygoid
**Supratemporals**			
Contact with supraoccipital	Present	Absent	Present
Posterior boundary	Beyond the posterior limit of braincase	Does not surpass the posterior limit of the braincase	Does not surpass the posterior limit of the braincase
**Quadrates**			
Shape in posterior view	About the same width in all extension	Approximately triangular, with ventral edge large and dorsal edge tapered	About the same width in all extension
Anterodorsal process	Present	Absent	Present
**Dentaries**			
Number of teeth	24	23	24
Location of dorsal process	14–24 tooth	16–23 tooth	16–24 tooth
Location of ventral process	14–21 tooth	16–21 tooth	16–21 tooth
Location of mental foramen	10–11th tooth	12–13th tooth	13th tooth
**Splenial**			
Size	About the same size of angular	Smaller than angular	About the same size of angular
Tapered process contacting dorsal portion of angular-splenial joint	Present	Absent	Absent
**Compound bone**			
Prearticular crest	Slightly higher than surangular crest	Much higher than surangular crest	Much higher than surangular crest

*Distribution (*Fig 15*)*. *Xenopholis werdingorum* is associated with dry formations of the Chiquitanos dry-forests and reaching within the Pantanal wetlands [72]. Marques [71] mentioned that the specimen of *X*. *werdingorum*, illustrated in page 89 [72], came from Luiz Eduardo Magalhães Power Plant (ca. 09°45'22"S 48°22'23"W), municipality of Palmas, state of Tocantins, Brazil, and, as consequence, inadvertently expanded its distribution 1,100 km airline northeastern from the municipality of Poconé (16°15'24"S 56°37'22"W), state of Mato Grosso, Brazil, locality where the species was first described, illustrated in page 73 [70]. Powell [17] expanded the distribution of *X*. *werdingorum* to the region of Beni, Bolivia, and considered the record mentioned above to Palmas into the species corology. However, such mention was likely due to an error since there is no cataloged voucher of this species for this region in ZUEC or UFMT-R collections (the only collections with available material for this species in Brazil). In fact, this was confirmed to us (O.A.V. Marques pers. comm. to PP in July 2019). Thus, we exclude the Palmas (state of Tocantins, Brazil) record from the distribution of *X*. *werdingorum*.

## Discussion

### Phenotypic characters and species boundaries

The results obtained with the study of distinct and putatively non-correlated qualitative (color pattern, pholidosis, osteology, and male genital features) and quantitative (meristic and morphometric traits) morphological characters entirely corroborates the current taxonomy of the genus *Xenopholis*. The disjunct pattern of distribution of *X*. *scalaris* in the Amazon basin and in the Atlantic Forest, and the extensive distribution of the genus in South America would suggest additional cryptic species in this the genus [5]. Surprisingly, we found that the phenotypic characters analyzed here together with niche overlap analyses are congruent with the current taxonomy.

Each *Xenopholis*’ species present a unique combination of qualitative features in male genital morphology, skull and vertebral osteology, coloration, and pholidosis. However, we found that coloration and the number of postoculars are both polymorphic, at least for *X*. *undulatus*. Additionally, the vertebral morphology and the number of segmental scales (ventral and subcaudals) of *X*. *undulatus* and *X*. *werdingorum* are indistinguishable, which is contrasting to what is available in the literature [5, 29] *vs*. this study. Zaher (Fig 95) [39] briefly described and illustrated a few differences between the hemipenial morphology of *X*. *scalaris* and *X*. *undulatus*. In the case of *X*. *werdingorum*, Jansen [5] had access only to female specimens and could not prepare the hemipenis of this species, which is very distinct from the other two species of the genus. Thus, the additional data for *X*. *werdingorum* from our study provides key diagnostic characters not available in the literature to distinguish among all three species of this genus. The variation of male genitalia (capitulum length with respect to the body; Fig 6) observed in the hemipenial body in the specimens in the Amazon and the Atlantic forests highlighted the higher polymorphism in the coastal populations of *X*. *scalaris*. A similar pattern was recovered to *Epicrates cenchria*, which also is distributed to both ecoregions [46]. However, to access if this polymorphism is geographically structured, more samples representative of a wider geographical distribution in the Atlantic Forest are needed.

Several studies have previously corroborated the monophyly of *Xenopholis* [1, 2, 73, 74]. However, the position of the genus inside the family Dipsadidae has been very unstable, being recovered in several different clades or even distinct tribes and subfamilies. Based on osteological features and the hemipenial traits gathered herein, we find no particular similarities among *Xenopholis* spp., *Caaeteboia amarali*, *Hydrodynastes* spp. (corresponding to a clade recovered in [1]). Nonetheless, we are aware that more morphological (and molecular) data from other species are necessary to test the phylogenetic position of *Xenopholis* within the family Dipsadidae. Therefore, the best solution to date is to consider the genus as Dipsadidae *incertae sedis*, awaiting for a robust phylogenetic hypothesis.

### Niche overlap and species delimitations

The distinct environmental niche space occupied by the three *Xenopholis’* species potentially indicate niche divergence as a mechanism for diversification of this genus, with each species adapted to a significantly distinct set of local environmental conditions (niche equivalence–Table 2) [75]. The uplift of some mountains in the central Brazilian Plateau and the expansion of open vegetation ecosystems since the Miocene have promoted both the diversification of organisms in these dry environments, as well as the geographical isolation of organisms in the disjunct forests [76, 77]. This pattern is supported by several other groups of snakes and lizards that also present this allopatric pattern along Amazonia, the South American Dry Diagonal (Caatinga, Cerrado, Chaco) and the Atlantic Forest (e.g., snakes of the *Bothrops atrox* group, snakes of the genus *Epicrates*, lizards of the *Kentropyx calcarata* group) [78, 79]. Additionally, niche predictions for *X*. *werdingorum* also indicate that environmental conditions in western Cerrado, Pantanal basin, and Bolivian dry forests are distinct from the Cerrado core area, again supporting niche divergence as a possible mechanism of speciation. This Pantanal/Cerrado pattern is also very similar to the distribution of many other reptiles and amphibians in this region [80]. Furthermore, the niche overlap between *X*. *undulatus* and *X*. *werdingorum* indicates that both environmental conditions across the entire ranges (niche similarity) and among the localities (niche equivalence) of these two species are significantly low (Table 2). With the increase in molecular data availability, alternative mechanisms to explain diversification in these groups could be adequately tested, for example, niche conservatism versus niche divergence across geographical barriers [81].

The set of broad-scale bioclimatic variables used here may not necessarily capture the set of determinant environmental conditions for some species [82]. For example, the microclimatic niche space experienced by *X*. *undulatus* in gallery forests may not be very different from the one experienced by *X*. *scalaris* in the Atlantic Forest or Amazonia. Besides, elevation is the most important variable to explain the distribution of *X*. *undulatus* (S2 Table), indicating that essential characteristics of the environmental niche were not completely captured by the bioclimatic and soil layers used here [68]. On the other hand, if the edge effect is more intense in forest patches and in gallery forests of highly seasonal areas such as the Cerrado and Caatinga if compared to core forest biomes, the microclimate experienced by species in these two environments will be different. Moreover, gallery forests in high elevation areas in Cerrado tend to be narrower than in the lowlands [83], and the size of forest fragments is also known to influence their microclimate and vegetation structure [84]. Therefore, the bioclimatic variables used here may still correctly characterize the broad-scale geographical distribution of these species [85]. Yet, studies on the specific microhabitat requirements for these snakes and the characterization of microenvironments in gallery forests versus forest biomes are necessary to test these alternative ideas.

The relictual or disjunct distributions of several taxa in South America suggests the role of past climate conditions in their current distribution [86]. Our results indicate that during the LGM, highly suitable areas for *X*. *undulatus* were more widespread, connecting portions of the range that are currently isolated in the Caatinga domain. On the other hand, for *X*. *scalaris*, highly suitable areas are more widely distributed under the current warmer temperatures than in the LGM (Fig 7). Past connections between Amazonia and Atlantic Forest have been proposed to explain the distribution of several taxa [79, 87–92]. In fact, the putative past forest bridges connecting Amazonia with coastal Atlantic Forest have returned to biogeographical debate [93–95]. However, in the case of *X*. *scalaris*, neither the current or the LGM predictions indicates highly suitable areas connecting these two domains, which may suggest that an accidental dispersal—rather than biome connectivity—might explain the disjunction. Interestingly, the overlap in the niche space occupied by these disjunct *X*. *scalaris* populations is not different from random, which indicates that beyond the current geographical isolation, these populations are evolving in distinct climatic spaces.

### Recent advance in snakes’ taxonomy and the past connections between Amazonia and Atlantic forest

The refuges theory was conceived in order to explain the great richness of species along the Amazonian lowlands and a speciation pattern in the absence of classical barriers [96, 97]. The main assumption of this theory proposes that the geographical differentiation of rainforest species results from the habitat isolation into distinct wet refuges during paleoclimatic cycles of glaciation throughout the Pleistocene. Later, with the amelioration of the climate in the wetter part of the dry cycle, the refuges coalesced again into continuous rainforest, and formerly isolated populations were brought into contact [98]. Those populations had then lost reproductive compatibility due to pre-zygotic reproductive isolation [97, 98]. Despite the predictions of early refuge model, paleopalynological data together with recent molecular estimates of divergence times between rainforest taxa have suggested pre-Pleistocene diversification in Amazonia [79, 88–92]. Such connections may explain the current corology of a multitude of species whose ranges encompass both forested regions, but not the open surrounding areas from the dry diagonal belt that separate them.

Recent advances in molecular datasets and analyses have enabled new hypotheses about space-time connections between Amazonia and Atlantic Forest. For instance, most of the discussion has been centered on the spatial routes and timing of former historical connections between such forested regions [91–94, 99, 100]. On the other hand, the ‘refuges theory’ could be employed by means of a reverse rationale, where historical forested connections in South America allowed maintenance (not differentiation) of gene flow between subpopulations of some species until recent times. Recently, this author [93] found more fragmentation of suitable habits for rainforest mammals during interglacial and present times than in the last glacial maximum (LGM). In addition, this author [93] detected expansion of suitable climatic conditions onto emerged continental shelf during LGM, which would have allowed forest restricted taxa to expand.

Regardless of reasons and processes involved, such disjunctive pattern between Amazonia and Atlantic Forest has emerged from disparate snake groups undergone detailed taxonomic reviews, as such: *Dipsas catesbyi* [101–103]; *Chironius carinatus* and *Chironius fuscus* [78]; *Liophis taenigaster* [104]; *Lachesis muta* [105]; *Epicrates cenchria* [46]; *Drymoluber dichrous* [106]; *Cercophis auratus* [107]; and *Xenopholis scalaris* (present study). There are possibly many more snake species sharing similar disjunct ranges, if we consider taxa still not assessed in detail through representative samples and/or based on the analysis of several character systems (i.e., phenotype and molecular evidence), including: *Imantodes cenchoa*, *Sibon nebulata* and *Siphlophis compressus* [13, 23]; *Thamndynastes pallidus* [108]; *Spilotes sulphureus* [109]; *Xenodon rhabdocephalus* [110]; and *Bothrops bilineatus* [95]. On the other hand, many of these taxa can be potentially separated in more than one species restricted to a single ecoregion, as occurred with *Chironius multiventris + Dendrophidion dendrophis* (restricted to Amazonia) and *Chironius foveatus + Dendrophidion atlantica* (restricted to Atlantic Forest) [111, 112]. The pattern from snakes’ distribution (including sister species), still enlightening to reinforce a recent contact with faunistic exchanges between Amazonia and Atlantic Forest.

Finally, the strong signature of the Amazonia-Atlantic Forest contact recovered in varied groups with disparate phylogenetic position (e.g., Boidae, Viperidae and several Dipsadidae tribes), unequal population densities (e.g., *Cercophis auratus* [low] vs. *Chironius fuscus* [high]), different reproductive modes (oviparous in *Lachesis muta* vs. viviparous *Bothrops bilineatus*) and distinct lifestyles (e.g., terrestrial, semi-arboreal and arboreal) suggest that snakes are good models to understand ancient land connection in forested environments by a unique combination of key ecological factors. We speculate herein that such a pattern may due to their reduced dispersal capacity (compared with other vertebrate groups such as mammals and birds), added to a higher resilience competence in relatively preserved areas. The snakes’ persistence in forested habitats was probably related to their unparalleled ability of exploring overlapping niche axes, associated to proper lifestyle, through particular foraging and thermoregulation strategies (e.g., arboreal gastropod-eating active forager specialists such as *Dipsas catesbyi* vs. arboreal ambush generalist as *Bothrops bilineatus*), and low metabolic costs as a rule [113, 114]. Most of these key factors related to putative resilience ability (e.g., foraging and thermoregulation strategies) are particularly sensitive to deforestation, which therefore impacts rainforest snake communities seriously and, in certain cases, may lead to species extinctions.

## Supporting information

S1 Fig Lateral (A) and dorsal (B) views of the head and dorsal (C) and ventral (D) views of the body of *Xenopholis scalaris* after preservation (MNRJ 26158) from Reserva Extrativista Ararixi, comunidade Manitã, Boca do Acre, state of Amazonas, Brazil. (DOCX)

S2 Fig Lateral (A) and dorsal (B) views of the head and dorsal (C) and ventral (D) views of the body of *Xenopholis undulatus* (MPEG 20526) from Santo Amaro Farm, Urbano Santos, state of Maranhão, Brazil. (DOCX)

S3 Fig Lateral (A) and dorsal (B) views of the head and dorsal (C) and ventral (D) views of the body of *Xenopholis werdingorum* (UFMT-R 12051) from Santo Antônio do Leverger, state of Mato Grosso, Brazil. (DOCX)

S1 Table List of modelling methods used for the ensembling prediction and the respective references. (DOCX)

S2 Table Variable importance calculated with AUC-based permutations from random forest models for each possible set of three environmental variables (median values per variable across all rounds). Asterisks* indicate selected six variables for the final model of each species.
(DOCX)

S1 Appendix (DOCX)

S1 Data (XLSX)

S2 Data (XLSX)

S3 Data (XLSX)
