# Supplementary material for: Taxonomic revision of the genus Xenopholis Peters, 1869 (Serpentes: Dipsadidae): Integrating morphology with ecological niche
Source: PLoS One. 2020 Dec 11;15(12):e0243210. doi: 10.1371/journal.pone.0243210 (PMC7732082; doi:10.1371/journal.pone.0243210)
Supplement: S2 Fig — (DOCX) [file pone.0243210.s002.docx]

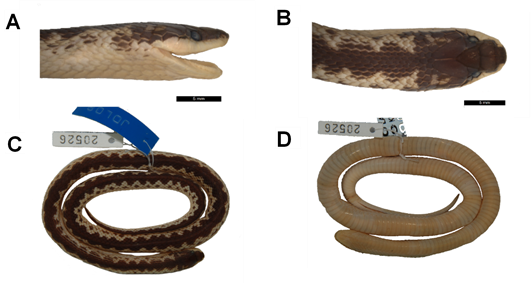


**S2 Fig.** Lateral (A) and dorsal (B) views of the head and dorsal (C) and ventral (D) views of the body of *Xenopholis undulatus* (MPEG 20526) from Santo Amaro Farm, Urbano Santos, state of Maranhão, Brazil.
