# Supplementary material for: Taxonomic revision of the genus Xenopholis Peters, 1869 (Serpentes: Dipsadidae): Integrating morphology with ecological niche
Source: PLoS One. 2020 Dec 11;15(12):e0243210. doi: 10.1371/journal.pone.0243210 (PMC7732082; doi:10.1371/journal.pone.0243210)
Supplement: S1 Table — (DOCX) [file pone.0243210.s004.docx]

**S1 Table.** List of modelling methods used for the ensembling prediction and the respective references.

| Algorithms | References |
| --- | --- |
| BIOCLIM | Busby, J. R. BIOCLIM – a bioclimate analysis and prediction system. in Nature Conservation: Cost Effective Biological Surveys and Data Analysis (eds. Margules, C. R. & Austin, M. P.) 64–68 CSIRO, 1991. |
| Boosted Regression Trees (BRT) | Elith, J., Leathwick, J. R. & Hastie, T. A working guide to boosted regression trees. J. Anim. Ecol. 77, 802–813 (2008). |
| DOMAIN | Carpenter, G., Gillison, A. N. & Winter, J. DOMAIN: a flexible modelling procedure for mapping potential distributions of plants and animals. Biodivers. Conserv. 2, 667–680 (1993). |
| Generalized Linear Models in elastic NET model paths (GLMNET) | Friedman, J., Hastie, T. & Tibshirani, R. Regularization paths for generalized linear models via coordinate descent. J. Stat. Softw. 33, 1 (2010). |
| Flexible Discriminant Analysis (FDA) | Hastie, T., Tibshirani, R. & Buja, A. Flexible discriminant analysis by optimal scoring. J. Am. Stat. Assoc. 89, 1255–1270 (1994). |
| Generalized additive models (GAM) | Hastie, T. & Tibshirani, R. Generalized Additive Models. Monographs on Statistics & Applied Probability. Chapman and Hall/CRC 1, (1990). |
| Generalized linear models (GLM) | McCullough, P. & Nelder, J. A. Generalized linear models. Monographs on Statistics & Applied Probability. Chapman and Hall/CRC (1989). |
| Maxlike - Likelihood-based Maxent | Royle, J. A., Chandler, R. B., Yackulic, C. & Nichols, J. D. Likelihood analysis of species occurrence probability from presence-only data for modelling species distributions. Methods Ecol. Evol. 3, 545–554 (2012). |
| Mixture Discriminant Analysis (MAD) | Royle, J. A., Chandler, R. B., Yackulic, C. & Nichols, J. D. Likelihood analysis of species occurrence probability from presence-only data for modelling species distributions. Methods Ecol. Evol. 3, 545–554 (2012). |
| Multivariate Adaptive Regression Spline (MARS) | Friedman, J. H. & others. Multivariate adaptive regression splines. Ann. Stat. 19, 1–67 (1991). |
| Random Forests (RF) | Breiman, L. Random forests. Mach. Learn. 45, 5–32 (2001). |
| Recursive Partitioning (RPart) | Therneau, T., Atkinson, E. & Ripley, B. rpart: Recursive partitioning for classification, regression and survival trees. R Packag. version 3, (2005). |
| Multi-layer Perceptron (MPL) | Rumelhart, D. E., Hinton, G. E. & Williams, R. J. Learning internal representations by error propagation. (1985). |
| Support Vector Machine (SVM) | Vapnik, V. N. The Nature of Statistical Learning Theory. (1995). |
| Radial Basis Function (RBF) | Acosta, F. M. A. Radial basis function and related models: an overview. Signal Processing 45, 37–58 (1995). |
