# Supplementary material for: Taxonomic revision of the genus Xenopholis Peters, 1869 (Serpentes: Dipsadidae): Integrating morphology with ecological niche
Source: PLoS One. 2020 Dec 11;15(12):e0243210. doi: 10.1371/journal.pone.0243210 (PMC7732082; doi:10.1371/journal.pone.0243210)
Supplement: S2 Table — Asterisks* indicate selected six variables for the final model of each species. (DOCX) [file pone.0243210.s005.docx]

**S2 Table.** Variable importance calculated with AUC-based permutations from Random Forest models for each possible set of three environmental variables (median values per variable across all rounds). Asterisks* indicate selected six variables for the final model of each species.

| Bioclim code | Relative variable importance | | Variable descriptions |
| --- | --- | --- | --- |
|  | *X. undulatus* | *X. scalaris* |  |
| bio_1 | 0.06 | 0.12 | Annual Mean Temperature |
| bio_2 | 0.11* | 0.39* | Mean Diurnal Range |
| bio_3 | 0.09* | 0.15 | Isothermality |
| bio_4 | 0.15* | 0.20* | Temperature Seasonality |
| bio_5 | 0.06 | 0.16 | Max Temperature of Warmest Month |
| bio_6 | 0.06 | 0.21* | Min Temperature of Coldest Month |
| bio_7 | 0.07 | 0.35 | Temperature Annual Range |
| bio_8 | 0.11* | 0.13 | Mean Temperature of Wettest Quarter |
| bio_9 | 0.06 | 0.12 | Mean Temperature of Driest Quarter |
| bio_10 | 0.07 | 0.14 | Mean Temperature of Warmest Quarter |
| bio_11 | 0.05 | 0.15 | Mean Temperature of Coldest Quarter |
| bio_12 | 0.14 | 0.18* | Annual Precipitation |
| bio_13 | 0.11* | 0.12 | Precipitation of Wettest Month |
| bio_14 | 0.08 | 0.18* | Precipitation of Driest Month |
| bio_15 | 0.13* | 0.17 | Precipitation Seasonality |
| bio_16 | 0.11 | 0.12 | Precipitation of Wettest Quarter |
| bio_17 | 0.09 | 0.18 | Precipitation of Driest Quarter |
| bio_18 | 0.06 | 0.11 | Precipitation of Warmest Quarter |
| bio_19 | 0.07 | 0.13 | Precipitation of Coldest Quarter |
| clay | 0.08 | 0.19* | Percentage of clay in the soil |
| sand | 0.09 | 0.13 | Percentage of sand in the soil |
| elevat | 0.18 | 0.18 | Elevation in meters |
