# Supplementary material for: Taxonomic revision of the genus Xenopholis Peters, 1869 (Serpentes: Dipsadidae): Integrating morphology with ecological niche
Source: PLoS One. 2020 Dec 11;15(12):e0243210. doi: 10.1371/journal.pone.0243210 (PMC7732082; doi:10.1371/journal.pone.0243210)
Supplement: S1 Appendix — (DOCX) [file pone.0243210.s006.docx]

**S1 Appendix.**

Institutional abbreviations are as listed in Sabaj [29]. Countries are given in capitals and bold, states (alphabetically arranged) in capitals and bold, municipalities in italic and localities in plain text. Specimens for which we prepared the hemipenis are listed with an asterisk.

***Xenopholis scalaris* (N = 261). BRAZIL:** IBSP 69145, UFAC 199, PUC-MG 4402, ZUEC 3874; **ACRE:** *Cruzeiro do Sul*: Parque Nacional Serra do Divisor Igarapé Ramon (UFAC 62, UFAC 79, UFAC 80); *Manoel Urbano*: Parque Estadual Chandles (UFAC 593); *Rio Branco*: São Raimundo farm, left bank of the Acre River (UFAC 251); **AMAPÁ:** *Serra do Navio*: Projeto Amaparí (MPEG 19677); **BAHIA:** *Almadina* (MUZUESC 10649, MUZUESC 11444, MUZUESC 11445, MUZUESC 13473, MUZUESC 13474*); *Barro Preto* (MUZUESC 531); *Cachoeira*: Pioneira road (MBML 1813); *Camacã* (MUZUESC 4697, MUZUESC 4698, MUZUESC 4699, MUZUESC 5992, MUZUESC 8453, MUZUESC 9068, MUZUESC 8385, MZUSP 20078); *Camamu* (CZGB 893, MUZUESC 4717, MUZUESC 4855, MUZUESC 5465); *Canavieiras* (CZGB 1107;CZGB 1089*); *Coarici* (MUZUESC 17745); *Ibirapitanga* (MUZUESC 4538); *Ilhéus* (CZBG 556, CZBG 2584, CZBG 3323, CZBG 351, CZBG 917, CZBG 725, CZBG 2956, CZBG 724, CZBG 84, CZBG 460, CZBG 4838, CZBG 4557, CZBG 2689, CZBG 202, CZBG 4172, CZBG 2474, CZBG 377, CZBG 5647, CZBG 2585, CZBG 1570, CZBG 793*, MUZUESC 14468, MUZUESC 13989, MUZUESC 13083, MUZUESC 17584, MUZUESC 2574, MUZUESC 4764, MUZUESC 13983, MUZUESC 13984, MUZUESC 13985, MZUSP 9622, MZUSP 17982, MZUSP 17983, MZUSP 20648, MZUSP 21594, UFMG 1736); *Itabuna* (CZGB 5815, PUC-MG 4401); *Itacaré* (CZGB 228, CZGB 875, CZGB 1693, CZGB 2163, CZGB 5544, MUZUESC 4933); *Itajuípe* (CZGB 885, CZGB 1135); *Ituberá* (CZGB 5611, CZGB 7360, CZGB 7361, CZGB 8648*); *Jussari* (CZGB 4778, MUZUESC 1722, MUZUESC 4711, MUZUESC 10651, MUZUESC 10652, MUZUESC 12165); *Pau Brasil* (MUZUESC 8981); *Una* (CZGB 176, MUZUESC 6818, MUZUESC 7537, MZUSP 15857, MZUSP 15858); *Uruçuca* (CZGB 310, CZGB 311, MUZUESC 8408, MUZUESC 8409, MUZUESC 14034, MZUSP 19946, MZUSP 20647); **ESPÍRITO SANTO:** *Linhares* (MNRJ 23804); **MANAUS:** (MPEG 20341, MPEG 20344, MPEG 20342, MPEG 20343); *Autazes* (UFMG 2154); *Borba* (MZUSP 5892); *Carauari* (MPEG 16343); *Coari*: Urucu River (MPEG 22272, MZUSP 10531); *Humaitá*: Igarapé Belém (MZUSP 4409); *Manicoré*: Passo Formoso farm (MPEG 20963); *Maués*: Paraconi River (MPEG 23826, MPEG 23833); *Presidente Figueiredo*: Balbina, Hydroelectric Power Plant Uatumã River: (MPEG 17401, MPEG 17471, MPEG 17445, MPEG 17565); *Boca do Acre*: Reserva Extrativista Ararixi, Manitã community (MNRJ 26158); **MATO GROSSO:** (MNRJ 332, MNRJ 331); *Apiacás* (MZUSP 11180, MZUSP 11181, MZUSP 11195); *Aripuanã* (MZUFV 1817, MZUFV 1818, MZUFV 1847, MZUSP 11427); *Comodoro* (PUC-MG 5318); *Juruena* (MZUSP 11326); *Paranaita* (ZUEC 3443*); **PARÁ:** (MPEG 23464); *Almeirim*: Monte Dourado (MPEG 21464), Rebio Maicuru (MPEG 23722); *Ananindeua*: Lago Azul (MPEG 15431); *Aveiro*: near to “Brasília Legal” (MPEG 25747); *Altamira*: Belo Monte Hydroelectric Power Plant (MZUSP 21711, MZUSP 21712, MZUSP 21713, MZUSP 21714, MZUSP 21715); *Belterra* (IBSP 88304); *Canaã dos Carajás* (FUNED 1989); *Capitão Poço*: Paraquequara (MPEG 5447); *Itaituba* (MPEG 24607): APA Tapajós (MPEG 24574, MPEG 24575, MPEG 24575): Parque Nacional Amazônia (MPEG 25264, MPEG 25263, MPEG 25261, MPEG 24619, MPEG 24614): Mina do Palito (MPEG 24597); *Juriti*: Mutum (MPEG 24327): Barroso (MPEG 22503); *Marabá* (UFMG 1426): Vila União (MPEG 26563): Serra Norte Carajás (MPEG 16794); *Melgaço*: Caxiuanã (MPEG 23761, MPEG 20455, MPEG 19993, MPEG 24600, MPEG 22108); *Parauapebas* (MPEG 23234): Floresta Nacional de Carajás (MPEG 26718); *Portel*: Floresta Nacional de Caxiuanã (MPEG 23163, MPEG 23215, MPEG 22723); *Porto de Moz* (MZUSP 19616); *Santa Bárbara* (MPEG 23602); *Santarém* (MPEG 19069); **PERNAMBUCO:** *Cabo de Santo Agostinho*: Reserva Ecológica do Gurjaú (MNRJ 17070, MNRJ 17071, MNRJ 17072, URCA 3609); *Lagoa dos Gatos*: RPPN Pedra D’antas (URCA 5099*); *Jaqueira*: RPPN Frei Caneca (URCA 6210*, URCA 6211); **RIO DE JANEIRO:** *Angra dos Reis* (IVB 3188, IVB 3189); *Magé* (IVB 3522*); **RONDÔNIA:** *Chupinguaia* (PUC-MG 5738,PUC-MG 5739); *Guarajá-mirim*: Reserva Biológica Rio Ouro Preto (MPEG 19568): Parque Estadual Guajará-mirim (MPEG 20370, MPEG 20371); *Machadinho D’Oeste* (MZUSP 21494, MZUSP 21955, MZUSP 21954, MZUSP 21662, MZUSP 21661, MZUSP 21479, MZUSP 21466, MZUSP 21467); *Ji-paraná*: Nova Colina (MZUSP 8503*); *Porto Velho*: Jirau Hydroelectric Power Plant (MPEG 23985, MPEG 26095, MZUSP 20481, MZUSP 18562, MZUSP 20865, MZUSP 21106, MZUSP 20482, MZUSP 18105, MZUSP 20844, MZUSP 19752, MZUSP 19448, MZUSP 19449, MZUSP 22338, MZUSP 22339, MZUSP 20678, MZUSP 20677, MZUSP 20845, MZUSP 2846, MZUSP 19445, MZUSP 19446, MZUSP 19447, MZUSP 19694, MZUSP 19693, MZUSP 19692, MZUSP 20873, MZUSP 20874, MZUSP 20872, MZUSP 18597, MZUSP 18598, MZUSP 18596, MZUSP 18917, MZUSP 18915, MZUSP 18916): Samuel Hydroelectric Power Plant (MPEG 17935, MPEG 17963, MPEG 17815, MPEG 18143): Santo Antônio Hydroelectric Power Plant (MPEG 25667, MPEG 25658, MPEG 25669, MPEG 25665, MPEG 25668, MPEG 25663): Santa Bárbara (MZUSP 8713*); *Santa Cruz da Serra* (MZUSP 8498*); **SÃO PAULO:** *Bertioga* (IBSP 81403); *Varjão do Guaratuba*: Perequê River (MZUSP 1890). **ECUADOR:** PASTAZA: Loracachi (QCAZ 11758). **PERU:** MADRE DE DÍOS: *Cusco Amazónico* (MUSM 3243, MUSM 7391); *Camisea* (MUSM 23227): *La Convención* (MUSM 26118); *Manu* (MUSM 17602, MUSM 17603, MUSM 24162, MUSM, 24350): *Tambopata* (MUSM 24425); UCAYALI: Sinesha River (MUSM 3311); HUÁNUCO: Dantas (MUSM 3330); LORETO: *Maynas* (MUSM 22341, MUSM 29905); PASCO: *Balognesi* (MUSM 20067); ECHARATE: *Pozo Cashiriari* (MUSM 26116).

***Xenopholis undulatus* (N = 76). BRAZIL: ALAGOAS:** *Campo Alegre* (MZUSP 17287); **GOIÁS:** *Barro Alto* (PUC-MG 1483); *Caldas Novas* (MNRJ 7322, PUC-RS 8618); *São Domingos*: Caverna Passa Três (MZUSP 12795); *Luziânia* (MZUSP 17694, MZUSP 17697); *Goiânia* (MZUSP 1688); *Minaçu*: Serra da Mesa Hydroelectric Power Plant (PUC-RS 8577, PUC-RS 8253, PUC-RS 8251, PUC-RS 8249); *Niquelândia*: Serra da Mesa Hydroelectric Power Plant (MZUSP 20493, MZUSP 20484, MZUSP 20494, MZUSP 20485, MZUSP 20487, MZUSP 20489, MZUSP 20488, MZUSP 20492, MZUSP 20490, MZUSP 20491, MZUSP 20486, MZUSP 11033, MZUSP 20975, MZUSP 20980, MZUSP 20982, MZUSP 20984, MZUSP 20979, MZUSP 20977, MZUSP 20981, MZUSP 20978, MZUSP 20976, MZUSP 20983); **MARANHÃO:** *Balsas* (MNRJ 26327, UFMT-R 11314); *Porto Franco* (MPEG 16557); *Urbano Santos*: Santo Amaro Farm (MPEG 20526, MPEG 20527); **MINAS GERAIS:** (FUNED 2180*, FUNED 3312); *Araguari* (FUNED 1180); *Belo Horizonte*: Parque das Mangueiras (FUNED 41, FUNED 87, FUNED 255); *Comendador Gomes* (MZUFV 2251); *Cristina* (MZUSP 15104*, MZUSP 18017); *Itumirim* (PUC-MG 777); *João Pinheiro*: Fruta Danta Farm (MNRJ 17232); *Lavras* (FUNED 1459); *Machado* (MNRJ 22153); *Mariana* (MZUSP 1499); *Moeda* (FUNED 194); *Rio Parnaíba*: Coxupé Farm (MNRJ 18728); *Nova Ponte* (FUNED 746, FUNED 763, FUNED 1136); *Oliveira* (FUNED 585); *Patrocinio* (PUC-MG 3827); *Riachinho* (MZUFV 1903); *Salto da Divisa* (MUZUESC 3604); *Serro* (FUNED 3124); *Uberlândia*: Miranda Hydroelectric Power Plant (FUNED 1948, PUC-MG 602); *Rio Preto*: Queimado Hydroelectric Power Plant (PUC-MG 1008); *Viçosa*: Campus Florestal (UFMG 1727, UFMG 1728, UFMG 1729); **MATO GROSSO:** *Campinápolis* (UFMT-R 11461); *Poconé*: Pouso Alegre Farm Hotel (UFMT-R 2106); **PARÁ:** *Marabá*: Carajás: Serra Norte (MPEG 16498); **PARAÍBA:** *Areia* (IBSP 89418, MZUSP 9100); **PIAUÍ:** *Ribeiro Gonçalves* (MNRJ 20626); **TOCANTINS:** *Miracema do Tocantins* (MZUSP 13255).

***Xenopholis werdingorum* (N = 12).** **BRAZIL: MATO GROSSO:** (UFMT-R 4071) *Santo Antônio do Leverger* (UFMT-R 12051*); *Chapada dos Guimarães*: APM Manso (UFMT-R 666); *Vila Bela de Santíssima Trindade*: Parque Estadual Ricardo Franco (UFMT-R 11902); *Pontes e Lacerda* (UFMT-R 10442*); *Jaciara* (UFMT-R 11538); *Cáceres* (UFMT-R 1526); *Poconé* (ZUEC 890); **MATO GROSSO DO SUL:** *Corumbá*: Serra do Amolar, RPPN Acurizal (UFMT-R 1191, UFMT-R 1195, UFMT-R 1193*, UFMT-R 1194).
